# The clinical efficacy and safety of berberine in the treatment of non-alcoholic fatty liver disease: a meta-analysis and systematic review

**DOI:** 10.1186/s12967-024-05011-2

**Published:** 2024-03-01

**Authors:** Qilong Nie, Mingyang Li, Caiyang Huang, Yongwei Yuan, Qiuyan Liang, Xiaojun Ma, Tengyu Qiu, Jianhong Li

**Affiliations:** 1 The Eighth Clinical Medical College, Guangzhou University of Chinese Medicine, Foshan, 528051 Guangdong China; 2 Foshan Hospital of Traditional Chinese Medicine, Guangzhou University of Chinese Medicine, No. 6, Qinren Road, Chancheng District, Foshan, 528051 Guangdong China

**Keywords:** Berberine, NAFLD, Meta, Metabolism, RCT

## Abstract

**Background:**

Non-alcoholic fatty liver disease (NAFLD) is becoming increasingly prevalent worldwide, emerging as a significant health issue on a global scale. Berberine exhibits potential for treating NAFLD, but clinical evidence remains inconclusive. This meta-analysis was conducted to assess the efficacy and safety of berberine for treating NAFLD.

**Methods:**

This study was registered with PROSPERO (No. CRD42023462338). Identification of randomized controlled trials (RCTs) involved searching 6 databases covering the period from their initiation to 9 September 2023. The primary outcomes comprised liver function markers such as glutamyl transpeptidase (GGT), alanine transaminase (ALT), aspartate transaminase (AST), lipid indices including total cholesterol (TC), triglyceride (TG), low-density lipoprotein cholesterol (LDL-C) and high-density lipoprotein cholesterol (HDL-C), homeostasis model assessment for insulin resistance (HOMA-IR) and body mass index (BMI). Review Manager 5.4 and STATA 17.0 were applied for analysis.

**Results:**

Among 10 RCTs involving 811 patients, berberine demonstrated significant reductions in various parameters: ALT (standardized mean difference (SMD) = − 0.72), 95% confidence interval (Cl) [− 1.01, − 0.44], *P* < 0.00001), AST (SMD = − 0.79, 95% CI [− 1.17, − 0.40], *P* < 0.0001), GGT (SMD = − 0.62, 95% CI [− 0.95, − 0.29], *P* = 0.0002), TG (SMD = − 0.59, 95% CI [− 0.86, − 0.31], P < 0.0001), TC(SMD = − 0.74, 95% CI [− 1.00, − 0.49], *P* < 0.00001), LDL-C (SMD = − 0.53, 95% CI [− 0.88, − 0.18], *P* = 0.003), HDL-C (SMD = − 0.51, 95% CI [− 0.12, 1.15], *P* = 0.11), HOMA-IR (SMD = − 1.56, 95% CI [− 2.54, − 0.58], *P* = 0.002), and BMI (SMD = − 0.58, 95% CI [− 0.77, − 0.38], *P* < 0.00001). Importantly, Berberine exhibited a favorable safety profile, with only mild gastrointestinal adverse events reported.

**Conclusion:**

This meta-analysis demonstrates berberine's efficacy in improving liver enzymes, lipid profile, and insulin sensitivity in NAFLD patients. These results indicate that berberine shows promise as an adjunct therapy for NAFLD.

*Trial registration* The protocol was registered with PROSPERO (No. CRD42023462338). Registered on September 27, 2023

**Graphical Abstract:**

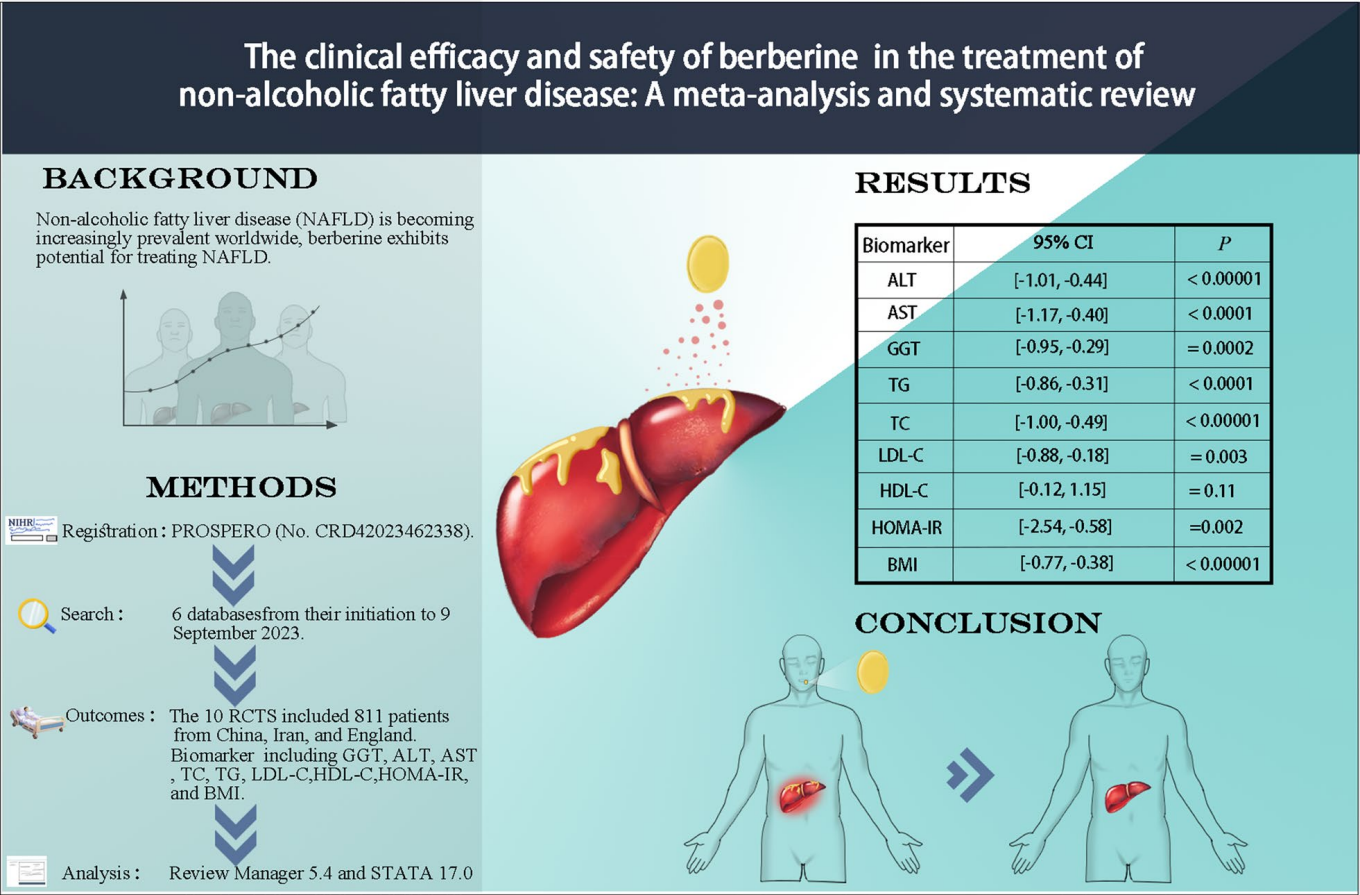

**Supplementary Information:**

The online version contains supplementary material available at 10.1186/s12967-024-05011-2.

## Background

Non-alcoholic fatty liver disease (NAFLD) is a clinicopathologic syndrome characterized by hepatic steatosis. This condition is often associated with metabolic comorbidities such as obesity, diabetes mellitus, and dyslipidemia [1]. The overall global prevalence of NAFLD was 38.2% from 2016 to 2019, and it has persistently increased over the past three decades [2]. Contrary to the initial perception of NAFLD as primarily affecting Western populations, it's worth emphasizing its elevated prevalence in North America, the Middle East, Asia, and numerous developing nations [3]. NAFLD typically exhibits no symptoms in its early stages, yet it carries the potential risk of progressing to cirrhosis and subsequently hepatocellular carcinoma, significantly impacting life expectancy [4].

The treatments of NAFLD can be divided into two categories: non-drug treatment strategies and medication-based treatments. Among non-drug treatments, lifestyle interventions stand out as a pivotal cornerstone therapy, with the regulation of glycolipid metabolism continuing to be the primary target in treating NAFLD [5]. Moreover, many drugs currently employed in clinical settings exhibit limited efficacy. Therefore, various novel drugs are still under development, represented by PPAR agonists, farnesoid X receptor agonists, and ethnopharmacological therapies [5].

Berberine is an odorless yellow powder, with a typical alkaloid bitter taste [6]. In China, the State Drug Administration has approved berberine for over-the-counter sale. Previous research has indicated that berberine enhances insulin sensitivity in patients, aiding in the regulation of blood sugar and lipid levels. Consequently, it finds application in clinical therapies for NAFLD [7–10].

The therapeutic efficacy of berberine in the treatment of NAFLD has been extensively validated through animal experimentation. In animal studies, the administration of berberine has the potential to enhance Sirtuin 1 expression, facilitate the deacetylation and stability of carnitine palmitoyl transferase 1A protein, and enhance liver fatty acid oxidation, thereby ameliorating NAFLD [11]. Additionally, Berberine alleviates NAFLD through intestinal microbiota—intestinal barrier—liver inflammation, and oxidative stress axis [12]. However, clinical studies have presented contradictory results regarding its efficacy. For example, in Nejati’s study [13], berberine failed to significantly lower the levels of lipids, fasting blood glucose, or liver enzymes in NAFLD patients. Another study [14] also reported no significant effect of berberine on HDL-C levels. Collectively, these findings indicate that berberine may have a limited influence on lipid metabolism in NAFLD patients. The meta-analysis, by pooling numerous studies, enlarges sample sizes, improves accuracy, and enhances statistical potency, thereby rendering the findings more compelling. Simultaneously, it can quantify and scrutinize the discrepant results across various studies, helping to discern whether the inconsistency is attributable to methodological heterogeneity or imperfections in the research data, such as small sample sizes, narrow age brackets, unequal gender representation, or technological limitations.

Therefore, the objective of this study was to assess the clinical efficacy and safety of berberine in the treatment of NAFLD through meta-analysis, aiming to provide more precise evidence for clinical decision-making.

## Methods

This meta-analysis was conducted according to Preferred Reporting Items for Systematic Reviews and Meta-Analyses (The PRISMA Statement [15]), and the protocol was registered with PROSPERO (No. CRD42023462338).

### Datasets and research technique

The following databases were included in this study: Wanfang Data; the Chinese National Knowledge Infrastructure; the Cochrane Central Register of Controlled Trials; Web of Science; Embase; and PubMed. Searches in these databases encompassed the entire period from their creation to September 9, 2023, with no language restrictions. The following search terms were used: ((((((((((nonalcoholic fatty liver) OR (nonalcoholic steatohepatitis)) OR (Non alcoholic Fatty Liver Disease)) OR (NAFLD)) OR (Nonalcoholic Fatty Liver Disease)) OR (Fatty Liver*, Nonalcoholic)) OR (Liver*, Nonalcoholic Fatty)) OR (Nonalcoholic Fatty Liver*)) OR (Nonalcoholic Steatohepatitis)) OR (Steatohepatitides, Nonalcoholic)) AND ("Berberine"[Mesh]) (Additional file 1: Table S1),

Independent searches were performed by two researchers in various databases employing specific keywords. Following this, a comparative analysis of the results was executed to ascertain completeness and accuracy. To encompass a wide range of relevant articles, citations from reviews on similar topics were also manually searched.

### Inclusion and exclusion criteria

The study’s eligibility criteria adhere to the PICOS framework (participants, interventions, comparisons, outcomes, and study design). The inclusion criteria are as follows: participants diagnosed with NAFLD (P). The experimental group received either berberine or a combination of berberine with other drugs (I). The control group received the same treatment as the experimental group excluding the berberine intake. (C). The article reports one or more of the following results: body mass index (BMI), total cholesterol (TC), triglycerides (TG), low-density lipoprotein cholesterol (LDL-C), high-density lipoprotein cholesterol (HDL-C), glutamyl transpeptidase (GGT), alanine transaminase (ALT), aspartate transaminase (AST), homeostasis model assessment for insulin resistance (HOMA-IR) (O). The study was conducted using a randomized controlled trial (RCT) methodology (S).

The exclusion criteria are as follows: (1) animal experiments, (2) reviews and case reports, (3) duplicate publications, (4) articles with incomplete data or that do not meet our specified requirements, and (5) individuals with alcoholic fatty liver disease or viral hepatitis.

### Data extraction

The data extraction and analysis were conducted independently by two evaluators. The extracted data comprised details such as the first author, publication year, total number of trial participants, respective numbers of experimental and control groups, intervention measures employed, and duration of the intervention.

Included outcomes are all expressed as mean ± SD: changes in BMI (kg/m^2^), TC (mmol/L), TG (mmol/L), LDL-C (mmol/L), HDL-C (mmol/L), GGT (U/L), ALT (U/L), AST (U/L), HOMA-IR.

### Quality assessment and risk of bias

The assessment of bias risk was independently conducted by two investigators using the Cochrane Collaboration’s Tool for Assessing Risk of Bias [16], classifying risk of bias as “high risk”, “low risk”, or “unclear risk”. The following terms were included in the Cochrane Collaboration’s Tool for Assessing Risk of Bias: selection bias, performance bias, detection bias, attrition bias, reporting bias, and other bias. These terms evaluated the methods employed for generating the randomization schedule and concealing treatment allocation, along with how blinding was implemented for participants, personnel, and outcomes. Additionally, we rigorously evaluated any indications of incomplete outcome data and selective reporting of outcomes; any disagreements were settled through discussions.

### Data synthesis and statistical analysis

#### Assessment and identification of heterogeneity

All analyses, completed by Review Manager (version 5.4) and STATA (version 17.0), presented results for continuous data as standardized mean difference (SMD) with a 95% CI. Heterogeneity between studies was estimated using the Higgins’ I^2^ test and stratified as follows: 0 ≤ I^2^ < 25%, “No heterogeneity”; 25% ≤ I^2^ < 50%, “Low heterogeneity”; 50% ≤ I^2^ < 75%, “High heterogeneity”; 75% ≤ I^2^, “Severe heterogeneity””. When I^2^ < 50%, a fixed-effect model was used for analysis, whereas I^2^ > 50%, a random-effect model was used for analysis.

Subgroup analysis and sensitivity tests were conducted to identify the sources of the heterogeneity. When I^2^ > 50%, subgroup analyses were conducted based on the total intake of berberine (< 100 g, 100–200 g, > 200 g), daily intake of berberine (< 1.5 g/d, = 1.5 g/d, > 1.5 g/d), duration of berberine intervention (< 4 months, = 4 months, > 4 months) and whether or not diabetes was combined (only NAFLD, NAFLD with Diabetes). In addition, sensitivity tests were performed by systematically removing one study at a time, aiming to reveal highly biased reports.

#### Assessment of publication bias

Given the tendency for papers with positive results to receive easier publication, our meta-analysis considered the impact of publication bias through the utilization of the funnel plot, the Egger linear regression test [17], and Begg’s test [18].

## Results

### Literature selection

**Fig. 1:**
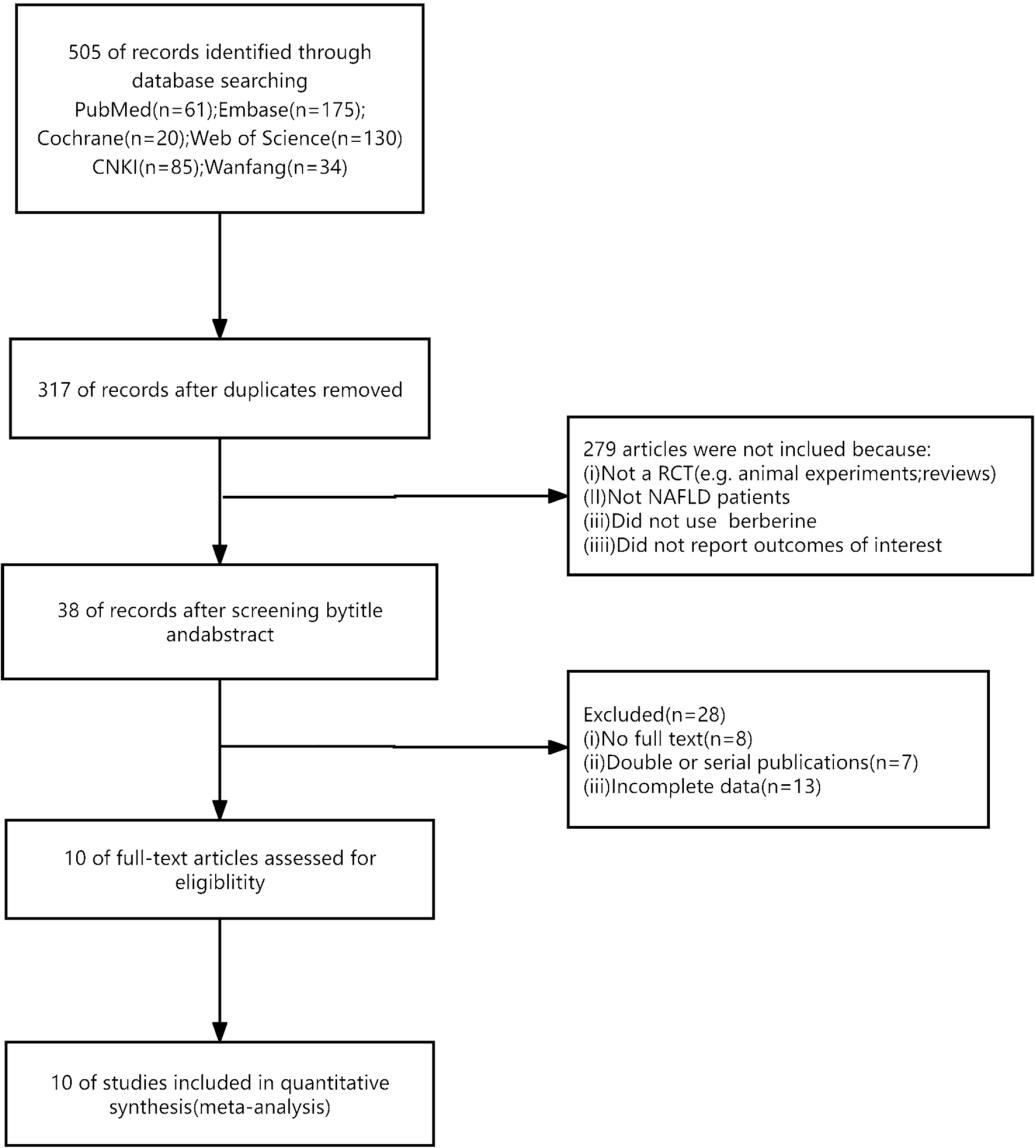
Flow diagram of the studies selection process

After implementing our research strategy, a total of 505 articles were obtained. Subsequently, following the removal of duplicates, 317 articles underwent screening based on their titles and abstracts. Among these, 279 articles were excluded due to failure to meet the inclusion criteria, which included not being randomized controlled trials (RCTs), not encompassing patients with NAFLD, lacking the utilization of berberine, or insufficient data for comprehensive reporting. Upon careful examination of the full texts of the remaining 38 articles, 28 articles were excluded due to reasons such as lack of full text (n = 8), double or serial publication (n = 7), and incomplete data availability (n = 13). Ultimately, a total of 10 articles [13, 14, 19–26] were included in this meta-analysis (Fig. 1).

### Study characteristics

A total of 10 RCTs involving 811 patients, conducted between 2010 and 2022, were included in this study. Among these studies, 8 RCTs [19–26] were carried out in China, while 2 RCTs [13, 14] in Iran and England. In 7 RCTs [14, 20–25], patients with NAFLD had comorbid diabetes, and metformin was administered to both the control and treatment group; while in the remaining 3 RCTs [13, 19, 26], the control group received lifestyle interventions. In 9 RCTs, the daily intake of berberine fluctuated between [14, 19–26] 0.6 g and 2 g, except for one study [13] where it was administered at 6.35 g/day. Berberine interventions ranged from a minimum of 7 weeks to a maximum of 24 weeks. Among these, 7 RCTs [19–22, 24–26] reported the diagnostic criteria for guidelines for the management of non-alcoholic fatty liver disease: an updated and revised edition (revised in 2010 [27]) and guidelines for diagnosis and treatment of nonalcoholic fatty liver diseases (revised in 2006 [28]). The remaining 3 RCTs [13, 14, 23] did not provide diagnostic criteria for NAFLD (Table 1).

**Table 1:** Characteristics of the included studies

Study	Country	Sample size(T/C)	Sex (M/F)	Age (mean, range)	Diagnostic criteria	Intervention of experimental group	Intervention of control group	Dose of berberine	Duration (weeks)	Outcomes
Zhao et al. (2022)	China	80 (40/40)	50/30	T: 46.57 (33–70) C: 46.85 (35–72)	I	Berberine + metformin	Metformin	0.2 g tid	12	1, 2, 3, 4, 5, 12, 13
Hu et al. (2021)	China	118 (58/60)	64/54	T: 42.85 (31–54) C: 43.21 (30–56)	I	Berberine + metformin	Placebo + metformin	0.5 g tid	24	1, 2, 3, 4, 5, 6, 7, 8, 9, 10, 11, 14, 15, 16, 17, 18
Han (2017)	China	100 (50/50)	49/51	T: 69.8 (66–82) C: 69.5 (65–80)	I	Berberine + metformin	Metformin	0.2 g–0.4 g tid	8	1, 2, 3, 4, 5, 6, 12, 13, 16
Cao et al. (2012)	China	78 (40/38)	46/32	T: 52.23 (29–69) C: 51.26 (33–67)	II	Berberine + metformin	Metformin	0.5 g tid	16	1, 2, 3, 4, 5, 6, 7, 8, 10, 16, 17, 18
Bai et al. (2011)	China	68 (38/30)	39/29	T: 56.6 (35–79) C: 56.1 (32–82)	II	Berberine	LSI	0.5 g tid	12	1, 2, 3, 4, 5, 6, 8, 10, 16, 18
Stephen et al. (2021)	England	87 [32/29 (0.5 g bid)/26 (1 g bid)]	NR	T: 58 (40–75) C (0.5 g bid): 58 (26–75) C (1 g bid): 53 (27–72)	NR	Berberine	Placebo	0.5 g bid/1 g bid	18	2, 3, 5, 17, 18
Lida et al. (2022)	Iran	48 (24/24)	36/12	T: 42.2 (NR) C: 40.6 (NR)	NR	Berberine	LSI	6.35 g/day	7	1, 2, 10, 17, 18
Yan et al. (2015)	China	108 (53/55)	NR	T: 50.64 (NR) C: 50.72 (NR)	NR	LSI + berberine	LSI	0.5 g tid	16	1, 2, 3, 4, 5, 7, 10, 16, 17, 18
Cui (2016)	China	80 (40/40)	58/22	T: 51.59 (37–65) C: 50.85 (39–62)	I	Berberine + metformin	Metformin	0.5 g tid	16	1, 2, 4, 5, 6, 7
Ning et al. (2013)	China	44 (22/22)	24/20	T: NR (35–70) C: NR (35–70)	I	Berberine + metformin	Metformin	0.5 g tid	16	1, 2, 6, 7

T: treatment group; C: control group; M: male; F: female; NR: not reported; LSI: lifestyle intervention; Tid: *ter in die*; Bid: *bis in die*; W: week; I: guidelines for management of nonalcoholic fatty liver disease: an updated and revised edition (revised in 2010); II: guidelines for diagnosis and treatment of nonalcoholic fatty liver diseases (revised in 2006) 1: total cholesterol; 2: triglycerides; 3: glutamyl transpeptidase; 4: aspartate transaminase; 5: alanine transaminase; 6: fasting blood glucose; 7: glycated hemoglobin; 8: glycated hemoglobin; 9: Controlled attenuation parameters of the liver; 10: body mass index; 11: percentage of body fat; 12: interleukin-17; 13: human transforming growth factor-β; 14: waist hip rate; 15: visceral fat area; 16: homeostatic model assessment of insulin resistance; 17: high-density lipoprotein cholesterol; 18: low-density lipoprotein cholesterol

### Risk of bias assessment

**Fig. 2:**
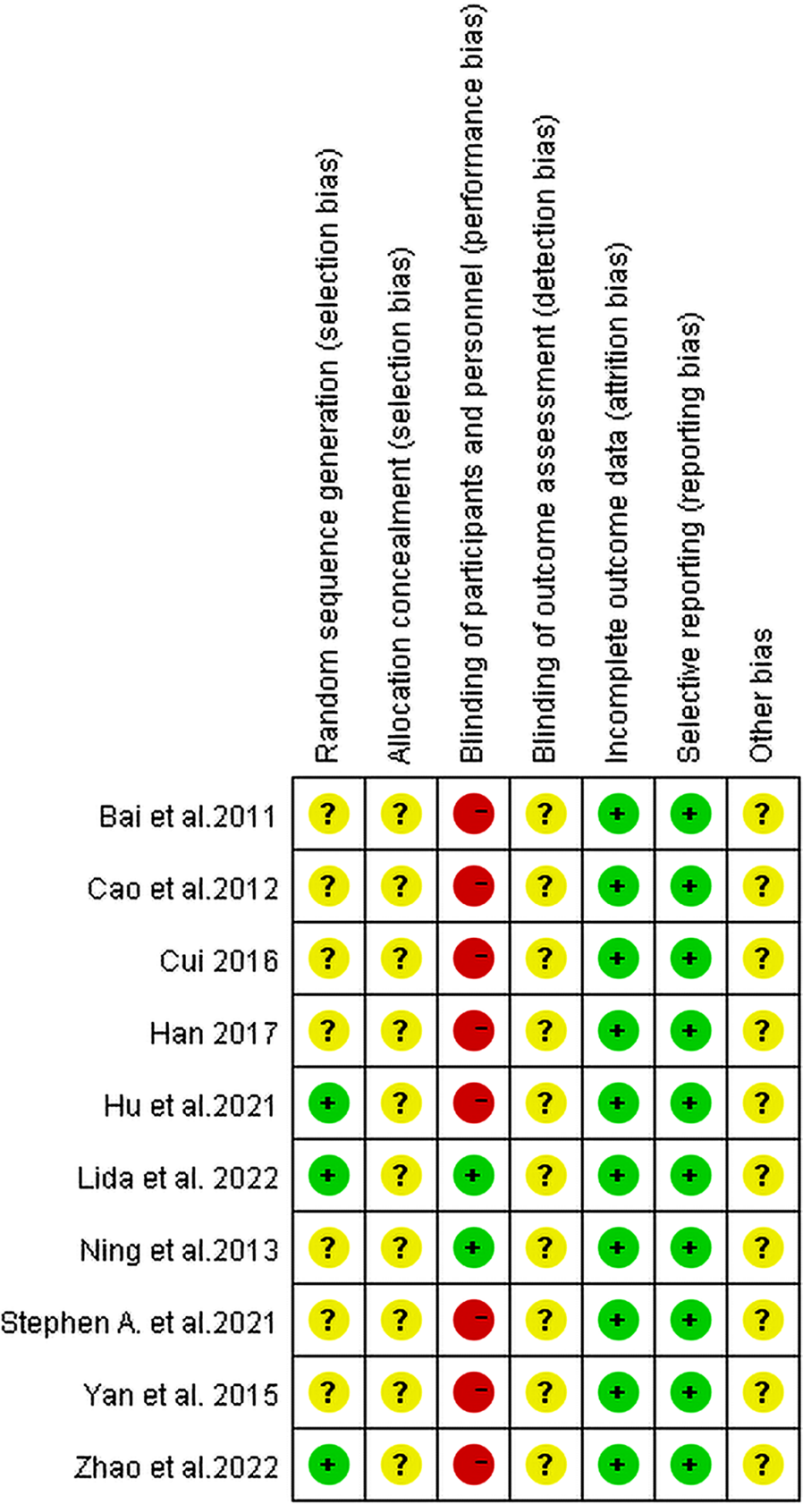
Assessment of risk of bias

The results of the risk of bias assessment of involved 10 studies are presented in Fig. 2. Among them, 3 studies [13, 19, 20] were categorized as low risk of bias due to their utilization of either the random numbers table or computer-generated random-allocation sequence for randomization. In contrast, the remaining 7 studies [14, 21–26] did not provide detailed methodology for randomization, resulting in an assessment of unclear risk. Notably, the allocation concealment was an unclear risk for all studies. Out of the reviewed studies, only two [13, 24] were deemed to exhibit a low risk of bias in terms of blinding, primarily because they adhered to the double-blinding principle. The rest of the studies [14, 19–23, 25, 26] were categorized as “high risk”. All the test results included in RCT were objective indicators, such as TC, TG, LDL-C, and HDL-C, etc. Therefore, detection bias was labeled as “low risk”. Regarding other biases, none of the studies provided adequate information for assessing whether there was a significant risk of bias and thus assessed as “unclear risk”.

### Effects of berberine on liver functions

#### Alanine transaminase

**Fig. 3:**
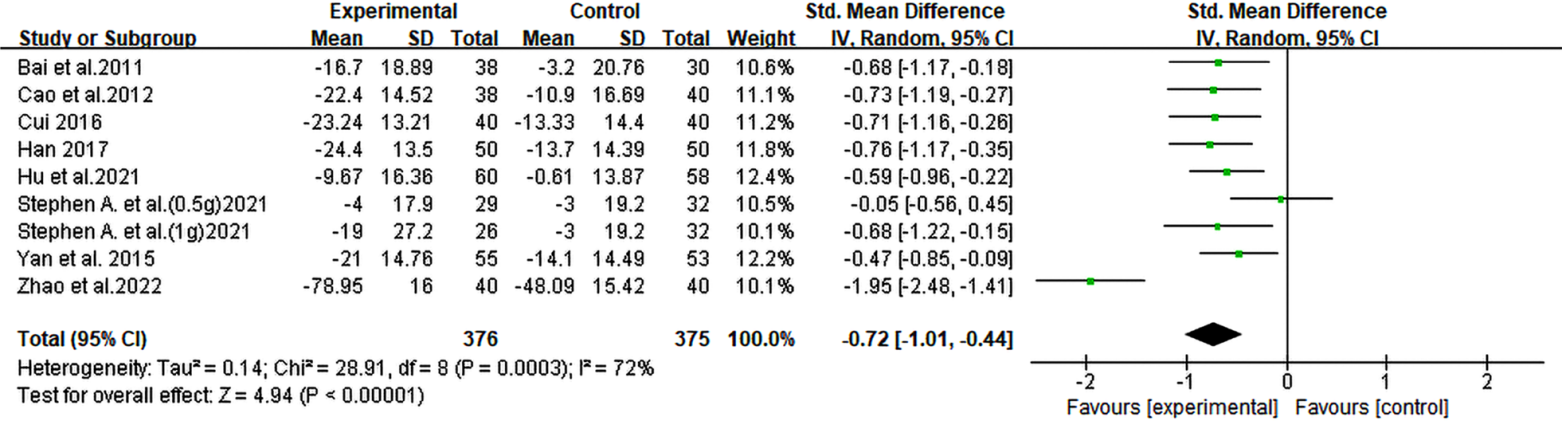
Forest plot for meta-analysis of ALT

A total of 8 RCTs [14, 19–23, 25, 26], involving 720 patients with NAFLD, were conducted to evaluate the levels of ALT biomarker. The meta-analysis results demonstrated that berberine exhibited significant efficacy in reducing ALT levels (SMD = − 0.72, 95% CI [− 1.01, − 0.44], *P* < 0.00001, I^2^ = 72%; Fig. 3). Subgroup analysis based on the duration of berberine intervention showed that heterogeneity was significantly diminished in the 4-month group (I^2^ = 0%). Moreover, a significant reduction in heterogeneity was also observed in the NAFLD with diabetes subgroup analysis (I^2^ = 3%) (Table 2, Additional file 2: Fig. S1).

**Table 2:** Subgroup analysis for outcomes

	Number of comparisons	Result SMD [95% CI]	*P-*value for overall effect	I^2^ (%)	*P*-value for subgroup differences
TG					
All comparisons	11	− 0.59 [− 0.73, − 0.45]	< 0.00001	73	
Total intake of berberine (g)					0.46
< 100	2	− 1.12 [− 2.39, 0.15]	0.08	93	
100–200	6	− 0.56 [− 0.76, − 0.37]	< 0.00001	0	
> 200	3	− 0.25 [− 0.92, 0.41]	0.45	82	
Daily intake of berberine (g/d)					0.21
< 1.5	3	− 0.88 [− 1.71, − 0.06]	0.04	89	
= 1.5	6	− 0.63 [− 0.81, − 0.45]	< 0.00001	0	
> 1.5	2	0.02 [− 0.73, 0.78]	0.95	74	
Duration of berberine intervention (months)					0.89
< 4	4	− 0.71 [− 1.53, 0.12]	0.09	91	
= 4	4	− 0.51 [− 0.74, − 0.29]	< 0.00001	0	
> 4	3	− 0.56 [− 0.82, − 0.30]	< 0.0001	0	
Whether or not diabetes was combined					0.68
Only NAFLD	3	− 0.78 [− 2.00, 0.44]	0.21	94	
NAFLD with diabetes	8	− 0.53 [− 0.68, − 0.37]	< 0.00001	0	
TC					
All comparisons	9	− 0.74 [− 1.00, − 0.49]	< 0.00001	63	
Total intake of berberine (g)					0.47
< 100	2	− 0.69 [− 1.15, − 0.23]	0.003	56	
100–200	5	− 0.89 [− 1.23, − 0.56]	< 0.00001	58	
> 200	2	− 0.39 [− 1.19, 0.41]	0.34	82	
Daily intake of berberine (g/d)					0.008
< 1.5	2	− 0.69 [− 1.15, − 0.23]	0.003	56	
= 1.5	6	− 0.87 [− 1.14, − 0.61]	< 0.00001	49	
> 1.5	1	0.05 [− 0.52, 0.61]	0.86	NA	
Duration of berberine intervention (months)					0.37
< 4	4	− 0.54 [− 0.92, − 0.16]	0.005	61	
= 4	4	− 0.94 [− 1.35, − 0.53]	< 0.00001	65	
> 4	1	− 0.77 [− 1.14, − 0.39]	< 0.0001	NA	
Whether or not diabetes was combined					0.41
Only NAFLD	3	− 0.56 [− 1.12, 0.01]	0.05	73	
NAFLD with diabetes	6	− 0.82 [− 1.11, − 0.53]	< 0.00001	61	
LDL-C					
All comparisons	7	− 0.53 [− 0.88, − 0.18]	0.003	74	
Total intake of berberine (g)					0.58
< 100	0	NA	NA	NA	
100–200	4	− 0.46 [− 0.99, 0.08]	0.1	81	
> 200	3	− 0.65 [− 1.10, − 0.20]	0.004	59	
Daily intake of berberine (g/d)					0.03
< 1.5	1	0.18 [− 0.35, 0.70]	0.51	NA	
= 1.5	4	− 0.73 [− 1.17, − 0.29]	0.001	76	
> 1.5	2	− 0.44 [− 0.91, 0.03]	0.07	29	
Duration of berberine intervention (months)					0.92
< 4	2	− 0.66 [− 1.56, 0.24]	0.15	82	
= 4	2	− 0.43 [− 1.02, 0.15]	0.15	74	
> 4	3	− 0.50 [− 1.19, 0.18]	0.15	83	
Whether or not diabetes was combined					0.72
Only NAFLD	2	− 0.66 [− 1.56, 0.24]	0.15	82	
NAFLD with diabetes	5	− 0.48 [− 0.90, − 0.06]	0.02	76	
HDL-C					
All comparisons	4	0.51 [− 0.12, 1.15]	0.11	82	
Total intake of berberine (g)					0.02
< 100	0	NA	NA	NA	
100–200	3	0.29 [− 0.34, 0.91]	0.37	82	
> 200	1	1.17 [0.77, 1.56]	< 0.00001	NA	
Daily intake of berberine (g/d)					0.08
< 1.5	0	NA	NA	NA	
= 1.5	3	0.70 [− 0.01, 1.42]	0.05	89	
> 1.5	1	− 0.12 [− 0.68, 0.45]	0.69	NA	
Duration of berberine intervention (months)					< 0.0001
< 4	1	− 0.02 [− 0.12, 0.08]	0.68	NA	
= 4	2	0.11 [− 0.10, 0.32]	0.32	92	
> 4	1	0.27 [0.19, 0.35]	< 0.00001	NA	
Whether or not diabetes was combined					0.08
Only NAFLD	1	− 0.12 [− 0.68, 0.45]	0.69	NA	
NAFLD with diabetes	3	0.70 [− 0.01, 1.42]	0.05	89	
ALT					
All comparisons	9	− 0.72 [− 1.01, − 0.44]	< 0.00001	72	
Total intake of berberine (g)					0.39
< 100	2	− 1.34 [− 2.50, − 0.18]	0.02	92	
100–200	5	− 0.53 [− 0.76, − 0.30]	< 0.00001	23	
> 200	2	− 0.62 [− 0.93, − 0.32]	< 0.0001	0	
Daily intake of berberine (g/d)					0.93
< 1.5	2	− 1.00 [− 2.85, 0.86]	0.29	96	
= 1.5	6	− 0.64 [− 0.81, − 0.47]	< 0.00001	0	
> 1.5	1	− 0.68 [− 1.22, − 0.15]	0.01	NA	
Duration of berberine intervention (months)					0.29
< 4	3	− 1.11 [− 1.86, − 0.37]	0.004	87	
= 4	3	− 0.61 [− 0.86, − 0.37]	< 0.00001	0	
> 4	3	− 0.45 [− 0.82, − 0.09]	0.01	46	
Whether or not diabetes was combined					0.26
Only NAFLD	2	− 1.31 [− 2.55, − 0.06]	0.04	91	
NAFLD with diabetes	7	− 0.58 [− 0.75, − 0.41]	< 0.00001	3	
AST					
All comparisons	7	− 0.79 [− 1.17, − 0.40]	< 0.00001	82	
Total intake of berberine (g)					0.1
< 100	2	− 1.33 [− 2.65, − 0.01]	0.05	93	
100–200	4	− 0.65 [− 0.91, − 0.40]	< 0.00001	22	
> 200	1	− 0.25 [− 0.62, 0.11]	0.17	NA	
Daily intake of berberine (g/d)					0.26
< 1.5	2	− 1.33 [− 2.65, − 0.01]	0.05	93	
= 1.5	5	− 0.56 [− 0.82, − 0.31]	< 0.0001	43	
> 1.5	0	NA	NA	NA	
Duration of berberine intervention (months)					0.08
< 4	3	− 1.20 [− 1.97, − 0.42]	0.003	87	
= 4	3	− 0.57 [− 0.82, − 0.32]	< 0.00001	4	
> 4	1	− 0.25 [− 0.62, 0.11]	0.17	NA	
Whether or not diabetes was combined					0.08
Only NAFLD	2	− 1.48 [− 2.52, − 0.44]	0.005	87	
NAFLD with diabetes	5	− 0.52 [− 0.72, − 0.32]	< 0.00001	17	
GGT					
All comparisons	8	− 0.62 [− 0.95, − 0.29]	0.0002	77	
Total intake of berberine (g)					0.24
< 100	2	− 1.15 [− 1.97, − 0.34]	0.005	84	
100–200	4	− 0.48 [− 0.77, − 0.19]	0.001	38	
> 200	2	− 0.32 [− 0.92, 0.27]	0.29	70	
Daily intake of berberine (g/d)					0.19
< 1.5	3	− 0.96 [− 1.55, − 0.37]	0.002	78	
= 1.5	4	− 0.36 [− 0.69, − 0.02]	0.04	61	
> 1.5	1	− 0.66 [− 1.21, − 0.12]	0.02	NA	
Duration of berberine intervention (months)					0.04
< 4	3	− 1.05 [− 1.55, − 0.55]	< 0.0001	71	
= 4	2	− 0.30 [− 0.62, 0.02]	0.06	17	
> 4	3	− 0.38 [− 0.78, 0.03]	0.07	54	
Whether or not diabetes was combined					0.04
Only NAFLD	2	− 1.22 [− 1.93, − 0.50]	0.0009	75	
NAFLD with diabetes	6	− 0.42 [− 0.66, − 0.17]	0.0009	46	
HOMA-IR					
All comparisons	5	− 1.56 [− 2.54, − 0.58]	0.002	96	
Total intake of berberine (g)					0.12
< 100	1	− 0.44 [− 0.83, − 0.04]	0.03	NA	
100–200	3	− 2.40 [− 4.45, − 0.35]	0.02	98	
> 200	1	− 0.75 [− 1.12, − 0.37]	< 0.0001	NA	
Daily intake of berberine (g/d)					0.03
< 1.5	1	− 0.44 [− 0.83, − 0.04]	0.03	NA	
= 1.5	4	− 1.91 [− 3.20, − 0.62]	0.004	96	
> 1.5	0	NA	NA	NA	
Duration of berberine intervention (months)					0.68
< 4	2	− 3.17 [− 8.58, 2.24]	0.25	99	
= 4	2	− 0.77 [− 1.57, 0.03]	0.06	86	
> 4	1	− 0.75 [− 1.12, − 0.37]	< 0.0001	NA	
Whether or not diabetes was combined					< 0.00001
Only NAFLD	1	− 5.96 [− 7.09, − 4.82]	< 0.00001	NA	
NAFLD with diabetes	4	− 0.68 [− 1.04, − 0.33]	0.0002	67	

#### Aspartate transaminase

**Fig. 4:**
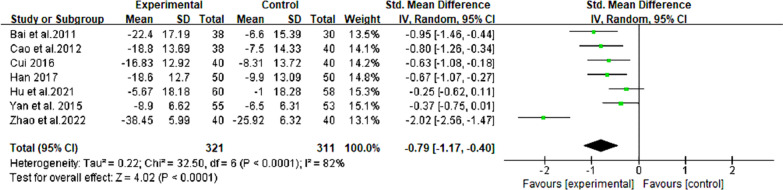
Forest plot for meta-analysis of AST

In 7 RCTs comprising 632 individuals with NAFLD, [19–23, 25, 26], berberine demonstrated efficacy in reducing AST levels, showing a remarkable reduction in the enzyme (SMD = − 0.79, 95% CI [− 1.17, − 0.40], *P* < 0.0001, I^2^ = 82%; Fig. 4). The subgroup analysis, specifically examining the duration of berberine intervention, revealed a significant reduction in heterogeneity within a specific group (= 4 months, I^2^ = 4%). Moreover, within the subgroup analysis of NAFLD patients with diabetes, heterogeneity was notably reduced to 17% (Table 2, Additional file 2: Fig. S2).

#### Glutamyl transpeptidase

**Fig. 5:**
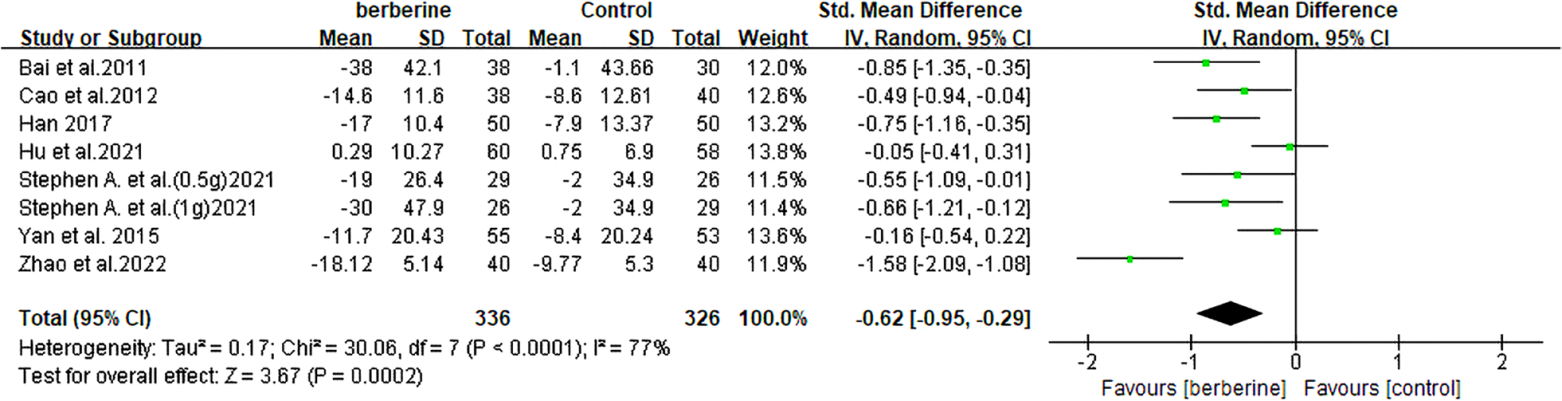
Forest plot for meta-analysis of GGT

A total of 659 patients with NAFLD were included in 7 RCTs [14, 19–21, 23, 25, 26], and the levels of GGT were assessed. The results showed that a significant decrease in GGT levels was noted when comparing the two groups (SMD = − 0.62, 95% CI [− 0.95, − 0.29], *P* = 0.0002, I^2^ = 77%; Fig. 5). Furthermore, analysis of the subgroup based on the duration of berberine intervention revealed a notable decrease in heterogeneity (= 4 months, I^2^ = 17%) (Table 2, Additional file 2: Fig. S3).

### Effects of berberine on lipid indices

#### Triglycerides

**Fig. 6:**
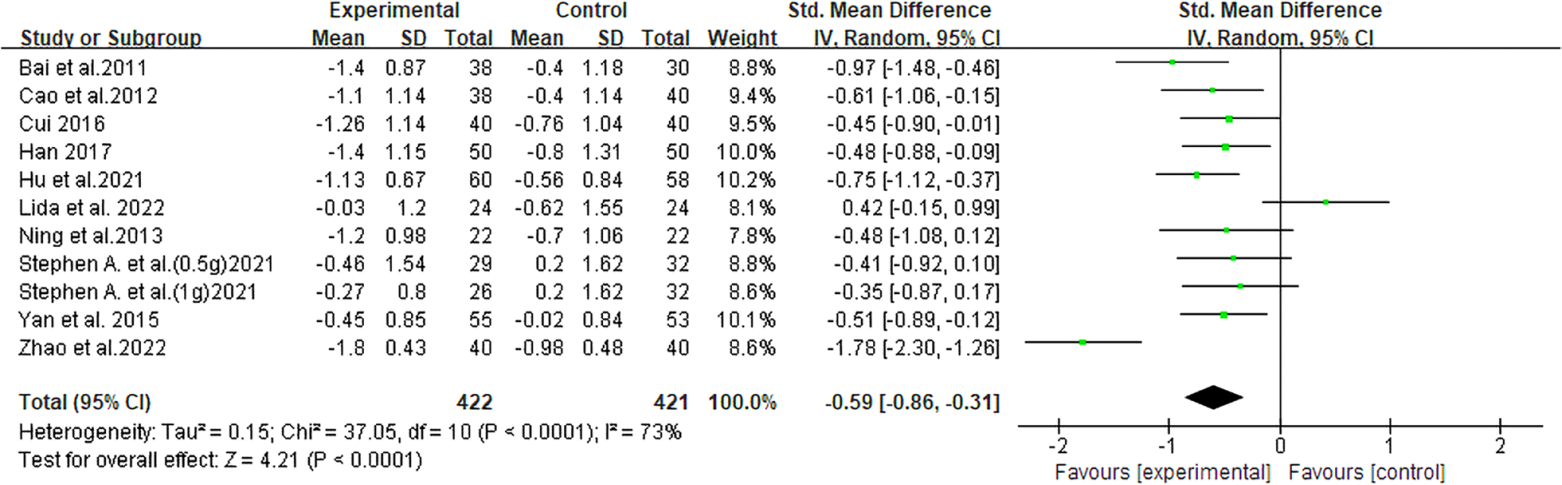
Forest plot for meta-analysis of TG

There were 10 studies [13, 14, 19–26] involving 811 patients that compared TG levels, and among these participants, 422 were in the experimental group and 265 were in the control group. The comprehensive analysis revealed that berberine exhibited potential in reducing TG levels in NAFLD patients (SMD = − 0.59, 95% CI [− 0.86, − 0.31], *P* < 0.0001, I^2^ = 73%; Fig. 6). Heterogeneity was effectively eliminated in all four subgroup analyses: total intake of berberine (100–200 g, I^2^ = 0%), daily intake of berberine (= 1.5 g/day, I^2^ = 0%), duration of berberine intervention (= 4 months, > 4 months, I^2^ = 0%), and whether or not diabetes was combined (NAFLD with diabetes, I^2^ = 0%)]. Notably, these subgroups excluded the two studies with polar extreme data [14, 18] (Table 2, Additional file 2: Fig. S4).

#### Total cholesterol

**Fig. 7:**
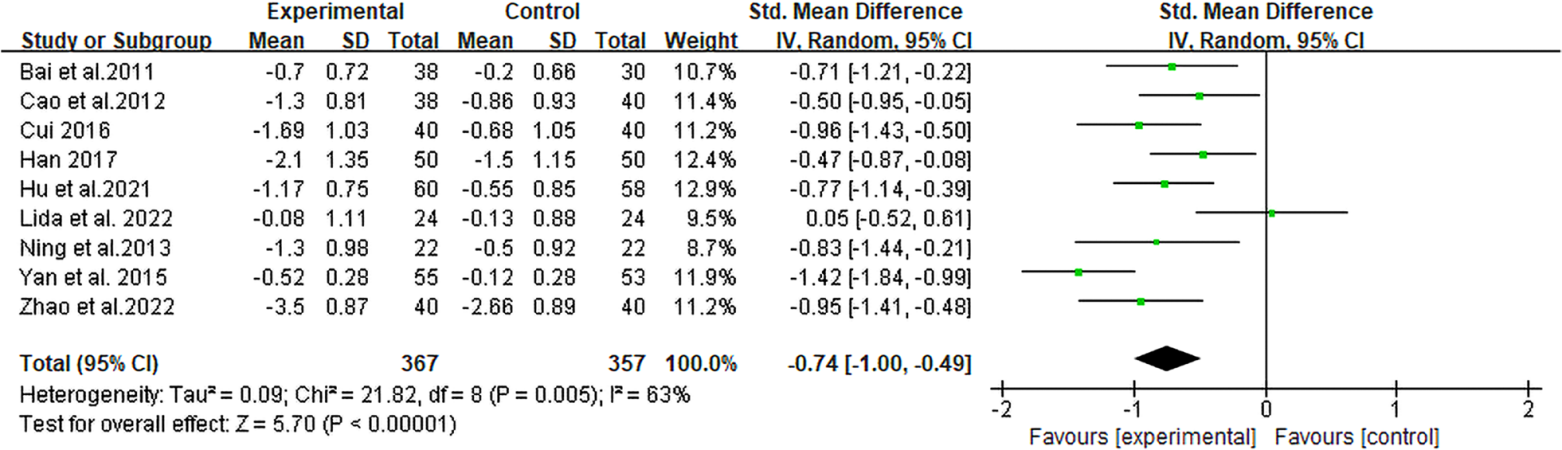
Forest plot for meta-analysis of TC

The TC levels were compared among 724 participants across 9 RCTs [13, 19–26]. Among them, 367 were in the experimental group and 357 were in the control group. The two groups exhibited a statistically significant disparity (SMD = − 0.74, 95% CI [− 1.00, − 0.49], *P* < 0.00001, I^2^ = 63%; Fig. 7). This indicated that TC levels in the experimental group were significantly lower than those in the control group. Additionally, no significant reduction in heterogeneity was observed in the subgroup analysis (Table 2, Additional file 2: Fig. S5).

#### Low-density lipoprotein cholesterol

**Fig. 8:**
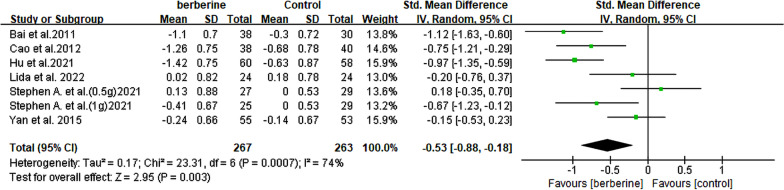
Forest plot for meta-analysis of LDL-C

Among the 6 RCTs [13, 14, 20, 23, 25, 26], a significant therapeutic effect on LDL-C was observed in 301 patients with NAFLD. The results demonstrated a significant reduction in LDL-C levels following berberine intervention (SMD = − 0.53, 95% CI [− 0.88, − 0.18], *P* = 0.003, I^2^ = 74%; Fig. 8). Of all the subgroup analyses, only the one focusing on daily berberine intake showed a significant decrease in heterogeneity (> 1.5 g/day, I^2^ = 29%) (Table 2, Additional file 2: Fig. S6).

#### High-density lipoprotein cholesterol

**Fig. 9:**
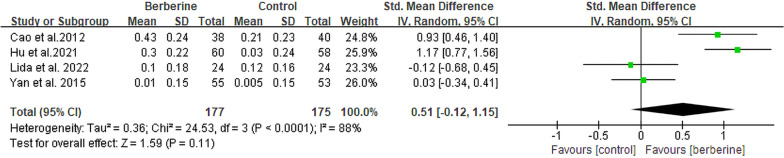
Forest plot for meta-analysis of HDL-C

The HDL-C levels were evaluated in 4 RCTs [13, 20, 23, 25] involving a total of 352 patients with NAFLD. Among these, two groups exhibited a significant statistical difference, indicating that berberine demonstrated superior therapeutic efficacy in increasing HDL-C levels (SMD = 0.51, 95% CI [− 0.12, 1.15], *P* = 0.11, I^2^ = 88%; Fig. 9). Besides, no substantial decrease in heterogeneity was observed across all four subgroup analyses conducted (Table 2, Additional file 2: Fig. S7).

### Effects of berberine on homeostasis model assessment for insulin resistance

**Fig. 10:**
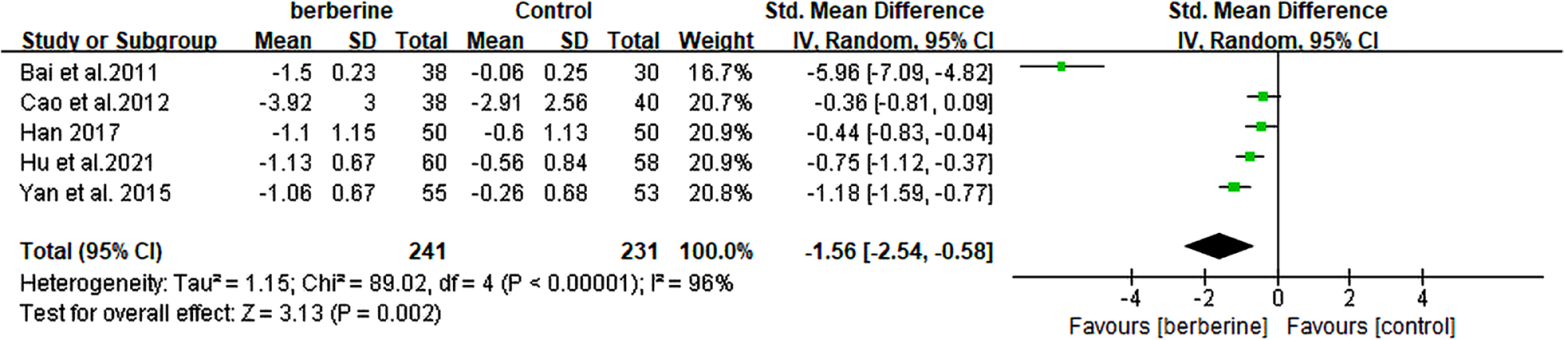
Forest plot for meta-analysis of HOMA-IR

The levels of HOMA-IR were meticulously monitored in a total of 472 patients diagnosed with NAFLD across 5 rigorously conducted RCTs [20, 21, 23, 25, 26]. The meta-analysis findings support the conclusion that berberine exhibited a potential for reducing HOMA-IR levels (SMD = − 1.56. 95% CI [− 2.54, − 0.58], *P* = 0.002, I^2^ = 96%; Fig. 10). In the subgroup analysis of NAFLD with diabetes, heterogeneity was effectively reduced to 67%, with no significant reduction observed in the other three subgroup analyses (Table 2, Additional file 2: Fig. S8).

### Effects of berberine on body mass index

**Fig. 11:**
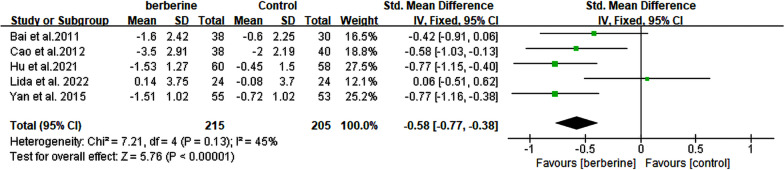
Forest plot for meta-analysis of BMI

A total of 5 RCTs [13, 20, 23, 25, 26] comprising 420 patients with NAFLD were included, and their BMI levels were reported. The meta-analysis demonstrated a significant improvement in BMI levels with the treatment of berberine (SMD = − 0.58, 95% CI [− 0.77, − 0.38], *P* < 0.0001, I^2^ = 45%; Fig. 11). Given the level of heterogeneity at 45%, subgroup analysis was omitted (Table 2).

### Adverse effects of berberine

A total of 5 RCTs [14, 19, 22, 24, 25] reported adverse effects including nausea, gastroesophageal reflux disease, constipation, etc. The statistics in Table 3 revealed that gastrointestinal reactions were the predominant adverse effects, with diarrhea and nausea being particularly prevalent. Besides, none of these symptoms were considered to be severe or irreversible. Indeed, all the adverse effects were resolved following appropriate symptomatic treatment.

**Table 3:** Adverse effects of berberine

Study	Experimental group (n=)	Adverse effects (n=)	Adverse reaction symptom
Zhao et al. 2022	40	6	Nausea (n = 2) Diarrhea (n = 2) Drowsiness (n = 2)
Cao et al. (2012)	38	9	Nausea (n = 3) Diarrhea Fatigue Constipation (n = 6)
Cui. (2016)	40	15	Dizziness (n = 3) Fatigue (n = 4) Nausea (n = 5) Diarrhea (n = 3)
Ning et al. (2013)	22	1	Gastrointestinal reaction
Harrison et al. (0.5 g) (2021)	33	9	Diarrhea (n = 4) Gastroesophageal reflux disease (n = 2) Nausea (n = 1) Headache (n = 2)
Harrison et al. (1 g) (2021)	34	15	Diarrhea (n = 9) Gastroesophageal reflux disease (n = 0) Nausea (n = 15) Headache (n = 1)

### Publication bias

**Fig. 12:**
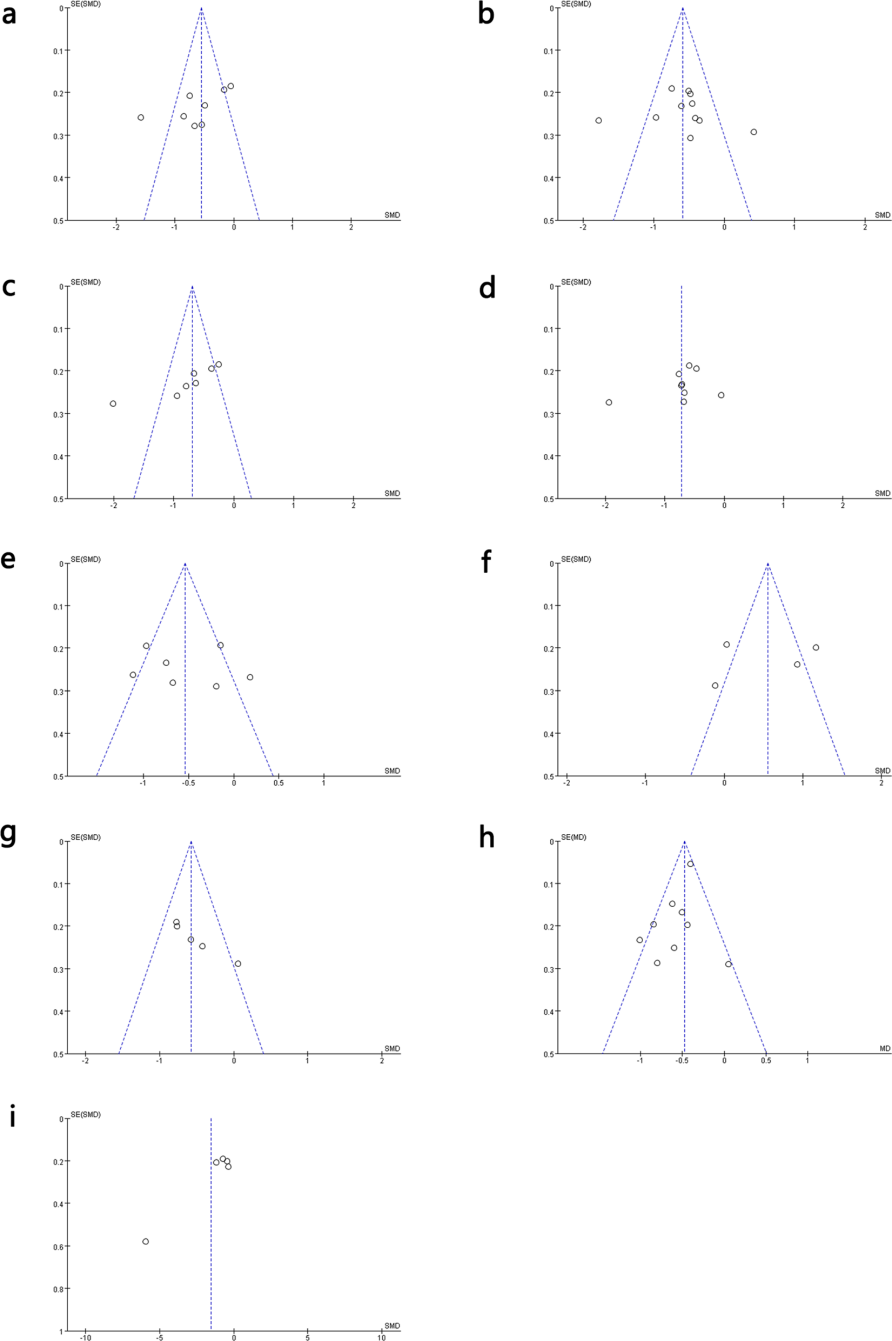
Funnel plots for assessing publication bias. **a** GGT; **b** TG; **c** AST; **d** ALT; **e** LDL-C; **f** HDL-C; **g** BMI; **h** TC; and **i** HOMA-IR

The funnel plot revealed asymmetry, but Begg and Egger’s tests did not reveal any significant bias (*P* > 0.05) in these results. Although Begg and Egger’s tests are more reliable for detecting potential bias in a larger pool of studies (usually more than 25), they were still important reference tools for this study (Fig. 12, Additional file 2: Fig. S9).

### Sensitivity analysis

To assess the robustness and reliability of the comprehensive findings in the meta-analysis, sensitivity analysis was employed. This method involved systematically removing individual studies and conducting a new meta-analysis with the remaining ones. We then examined whether the results exhibited significant discrepancies compared to those before exclusion, thereby ensuring the robustness of our findings. For all assessments, the results remained consistent following sensitivity analysis with the exclusion of included data. Specifically, data from eight studies were available for analysis of ALT levels. Upon exclusion of Zhao’s study, a notable decrease in heterogeneity was observed (I^2^ = 0%) (Additional file 2: Fig. S10). During our sensitivity analysis, despite the absence of a statistically significant change in HDL-C levels among NAFLD patients treated with berberine in the initial findings, a more detailed examination through sensitivity analysis highlighted the subtleties inherent in these results. This was particularly evident due to the discernible impact on the overall outcomes resulting from the exclusion of specific studies from the analysis. These observations underscore the paramount importance of meticulously considering variables such as study duration, quality, and participant characteristics when elucidating the implications of berberine on NAFLD patients.

## Discussion

### Summary of the main results

This meta-analysis of 10 RCTs with 811 patients provides evidence that berberine when employed as an adjunct therapy, can improve liver enzymes, dyslipidemia, insulin resistance, and body weight in patients with NAFLD while exhibiting minimal adverse effects. It is noteworthy that several outcomes demonstrated heterogeneity. The heterogeneity observed in certain indicators might be attributed to significant bias in individual studies, particularly in cases involving ALT, AST, and HOMA-IR. This can be explained through sensitivity analysis (Additional file 2: Fig. S10). Conversely, for the heterogeneity observed in lipid profiles, subgroup analysis, and sensitivity analysis failed to identify the sources, potentially stemming from initial variations in the measurement methods of each indicator.

In our subgroup analysis, we found that elevating the dosage of berberine did not yield a statistically significant improvement in its efficacy concerning lipid profiles. However, extending the duration of administration, particularly beyond 4 months, might be more beneficial for regulating lipid profiles. Conversely, regarding liver and kidney function indicators, a lower daily intake proved to be more effective in improving liver function indicators. Therefore, based on these results, it is suggested that a lower dose with long-term intake of berberine may confer more substantial benefits for patients with NAFLD in a clinical setting.

### Potential mechanisms of berberine in the treatment of NAFLD

NAFLD is characterized by hepatic lipid accumulation in individuals who do not consume significant amounts of alcohol. The pathogenesis of NAFLD is intricate and multifactorial. Key mechanisms include: (1) Insulin resistance, which leads to enhanced lipolysis in adipose tissue and increased influx of free fatty acids into the liver, contributing to hepatic steatosis; (2) Adipokine imbalance, with altered levels of adipokines such as elevated leptin and resistin, as well as decreased adiponectin, promoting inflammation, insulin resistance, and hepatic steatosis; (3) Oxidative stress causing increased reactive oxygen species and lipid peroxidation damage hepatocytes, and activating inflammatory pathways and stellate cells leading to fibrosis; (4) Dysbiosis in gut microbiota resulting in changes that increase intestinal permeability, facilitating the translocation of bacteria and bacterial products, thereby promoting hepatic inflammation; (5) Hepatic inflammation involving activation of Kupffer cells and recruitment of inflammatory cells releasing cytokines and chemokines that aggravates insulin resistance while causing hepatocyte injury [29, 30]. In summary, the development of NAFLD involves multiple parallel hits derived from adipose tissue, gut microbiota dysbiosis, and the liver itself. The interplay among these factors generates a hepatic environment characterized by pro-inflammatory and pro-fibrogenic processes, consequently precipitating steatosis, inflammation, and fibrosis.

Berberine improves insulin sensitivity by increasing the expression and enhancing the activation of insulin receptor (InsR) [31]. As shown by previous studies, berberine upregulates InsR expression via a protein kinase C-dependent mechanism. Moreover, berberine also improves insulin sensitivity by inhibiting protein tyrosine phosphatase 1B activity, thereby affecting the phosphorylation of InsR and insulin receptor substrate 1 (IRS-1) [32, 33]. Berberine alleviates insulin resistance by activating the Adenosine Monophosphate-activated protein kinase (AMPK) pathway in muscle and liver tissue, thereby enhancing glucose uptake and glycogen synthesis [31, 34, 35]. The HOMA-IR index serves as a pivotal parameter for assessing insulin sensitivity. Besides, HOMA-IR enables the quantification of insulin resistance and β cell function based on basal glucose and insulin concentrations, making it a widely utilized surrogate marker for assessing insulin resistance in research studies. In this meta-analysis, the administration of berberine resulted in a significant reduction in HOMA-IR among patients with NAFLD, as well as those presenting with concomitant diabetes.

Patients with NAFLD commonly exhibit significant dysregulation of serum lipid profile. The findings of this meta-analysis indicate that berberine can effectively regulate the levels of these biomarkers. Besides, intrahepatic TG accumulation indicates imbalanced hepatic energy metabolism and serves as a biomarker of NAFLD [36, 37]. The levels of intrahepatic TG are regulated by the equilibrium among hepatic lipid synthesis, decomposition, and excretion. Lipid synthesis involves a cascade of enzymatic reactions that convert acetyl-CoA into fatty acids, ultimately leading to TG production. Meanwhile, TG decomposition primarily occurs through mitochondrial β-oxidation of fatty acids, resulting in the generation of both heat and ATP. Additionally, the process of hepatic lipid synthesis commences with the generation of acetyl-CoA, serving as the fundamental precursor for fatty acid biosynthesis [38].

Berberine improves lipid metabolism in the liver through several mechanisms. One such mechanism involves the upregulation of microsomal triglyceride transfer protein [39], promoting the release of TG from liver cells into the bloodstream. This reduces TG accumulation in hepatocytes and alleviates hepatic steatosis. Berberine also increases the expression and enhances the activity of Adenosine Triphosphate-binding cassette transporter A1, which mediates the efflux of cholesterol and phospholipids from hepatocytes onto apolipoproteins to form HDL particles [40]. This facilitates cholesterol release from liver cells.

In addition, berberine enhances mitochondrial function through coordinated effects on energy metabolism, oxidative stress, and mitochondrial biogenesis. Moreover, the activation of transcription factors, such as peroxisome proliferator-activated receptor gamma coactivator 1-α, induced by berberine, promotes mitochondrial biogenesis through the up-regulation of gene expression associated with this process. Furthermore, Berberine also reduces mitochondrial reactive oxygen species (ROS) generation by activating sirtuin 3 [41]. In skeletal muscle, berberine promotes mitochondrial biogenesis, and the modulation of sirtuin 1 activity may also contribute to berberine’s mitochondrial effects [35, 42]. These mechanisms suggest that berberine exhibits efficacy in ameliorating lipid metabolism disorders associated with NAFLD.

In addition to lipid metabolism disorders, inflammation is also an important factor in the decline of liver function in NAFLD patients. Elevated levels of inflammatory cytokines such as tumor necrosis factor-α and interleukin-1β can induce liver cell damage through mechanisms involving oxidative stress, mitochondrial dysfunction, and apoptosis [43]. Damaged liver cells further exacerbate inflammation and impair liver function, initiating a vicious cycle that worsens hepatitis and leads to liver dysfunction [44]. Berberine suppresses inflammation by inhibiting phosphoinositide 3-kinase/protein kinase B and nuclear factor κ-light-chain-enhancer of activated B cells pathways involved in inflammatory responses while activating AMPK and nuclear factor erythroid 2-related factor (Nrf2) pathways known for the anti-inflammatory effects [45, 46]. Our study demonstrates that berberine exerts a dual effect on insulin sensitivity and liver function, effectively mitigating insulin resistance while significantly enhancing hepatic function by alleviating inflammation.

Other than regulating glucolipid metabolism and reducing inflammation, berberine also ameliorates NAFLD by modulating gut microbiota and alleviating oxidative stress. Berberine modulates the gut microbiota by enhancing the abundance of beneficial bacteria like Bifidobacterium and Lactobacillus while reducing opportunistic pathogens [47, 48]. Berberine can modulate the physiological axis connecting the gut and the liver, leading to a balanced composition of intestinal microbes, maintenance of intestinal integrity, and reduction in enterogenic endotoxins entering the liver. As a result, this multifaceted influence contributes to effectively reducing liver inflammation and steatosis.

**Fig. 13:**
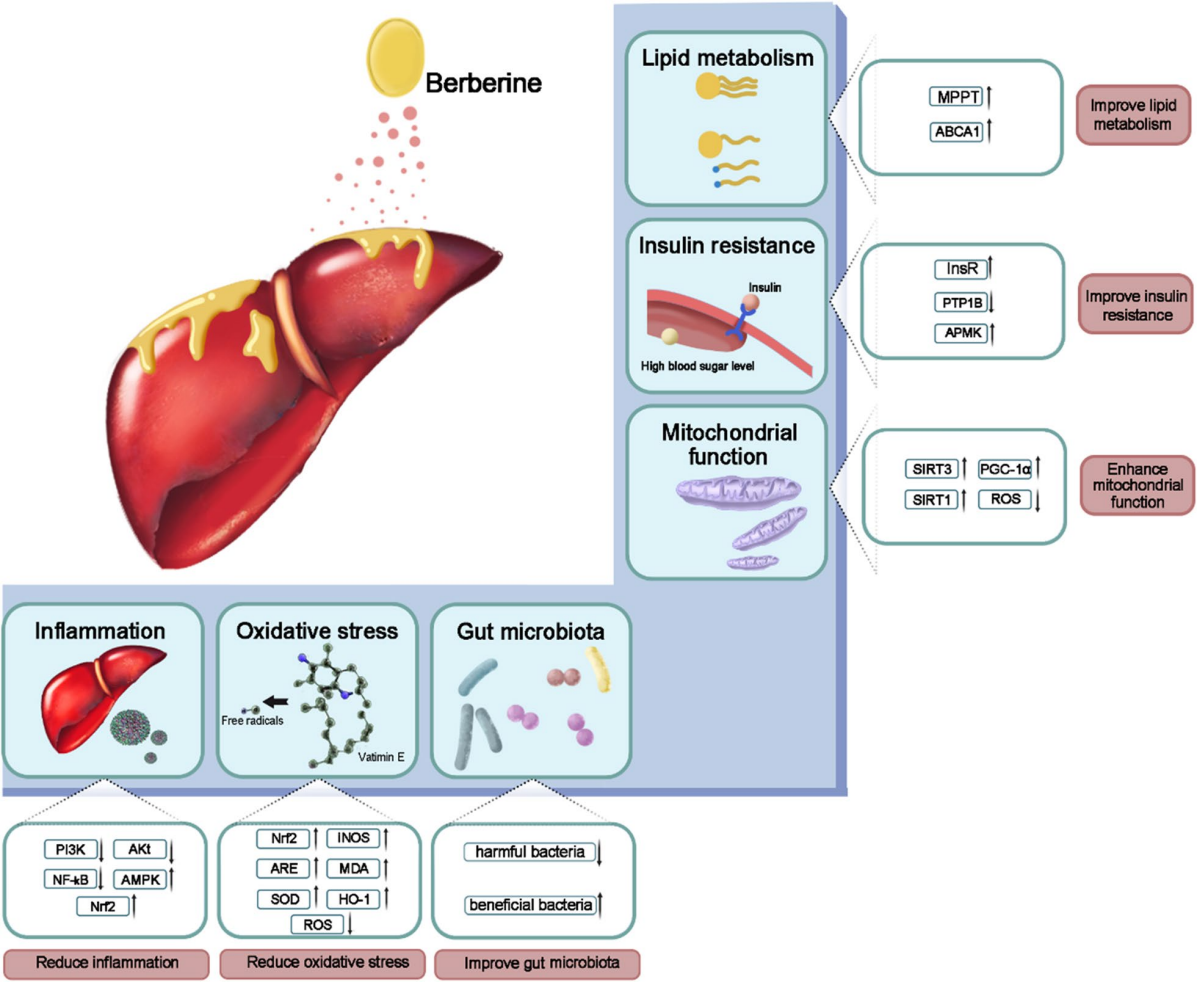
Molecular pathway mechanisms of berberine therapy for NAFLD

Berberine reduces oxidative stress in the liver by activating Nrf2 and antioxidant response element antioxidant pathway and increasing the expression of antioxidant factors like superoxide dismutase, inducible nitric oxide synthase, and heme oxygenase-1 [49, 50]. In previous studies, Berberine has demonstrated efficacy in clearing reactive oxygen species (ROS) and malondialdehyde. Through enhancing antioxidant defenses and reducing ROS accumulation, berberine protects against oxidative injury in the liver[51] (Fig. 13).

Berberine also exhibits excellent safety in both animal toxicology studies and demonstrates favorable safety profiles in clinical observations [52]. In our meta-analysis, the included study reported dosages ranging from 0.6 to 6.35 g. All observed adverse reactions were mild, and recovery from these reactions could be achieved through symptomatic treatment. Furthermore, the pharmacokinetic studies reveals a significantly higher concentration of berberine metabolites in the liver (50–70 times greater than plasma levels) following oral administration [53]. The distribution of berberine extends beyond the blood–brain barrier, with its metabolites exhibiting widespread presence in various organs including the liver, muscle, kidney, lung, heart, brain, pancreas, and adipose tissue [54]. Considering the extensive tissue absorption and broad pharmacological effects, berberine may hold significant therapeutic potential for the treatment of NAFLD, a multi-system metabolic disorder. This underscores its significance in addressing the condition.

### Quality of evidence

In our systematic review, we utilized the GRADEpro guideline development tool to rigorously evaluate the efficacy of berberine in the management of NAFLD. This evaluation integrated findings from 10 RCTs for each outcome, spanning a broad patient cohort. Our findings demonstrated a consistent benefit of berberine across several critical endpoints, including improvements in liver enzymes (GGT, AST, ALT), lipid profiles (LDL-C, HDL-C, TC, TG), BMI, and HOMA-IR, as evidenced by SMD ranging from moderate to substantial effect sizes (0.51 to 1.56). The quality of evidence for each outcome was diligently assessed based on GRADE criteria, addressing concerns related to the risk of bias, inconsistency, indirectness, and imprecision. The high quality of evidence (⊕⊕⊕⊕) across all outcomes suggests a robust confidence level in the effect estimates, reinforcing the potential of berberine as a significant therapeutic intervention in NAFLD (Table 4).

**Table 4:** Quality of evidence: berberine compared to control treatment for NAFLD

Quality assessment							No of patients		Effect		Quality	Importance
No of studies	Design	Risk of bias	Inconsistency	Indirectness	Imprecision	Other considerations	Berberine	Control	Relative (95% CI)	Absolute		
**GGT (better indicated by lower values)**												
8	Randomised trials	No serious risk of bias	No serious inconsistency	No serious indirectness	No serious imprecision	None	336	326	–	SMD 0.62 lower (0.95 to 0.29 lower)	⊕⊕⊕⊕ HIGH	IMPORTANT
**TG (better indicated by lower values)**												
11	Randomised trials	No serious risk of bias	No serious inconsistency	No serious indirectness	No serious imprecision	None	422	421	–	SMD 0.59 lower (0.86 to 0.31 lower)	⊕⊕⊕⊕ HIGH	IMPORTANT
**AST (better indicated by lower values)**												
7	Randomised trials	No serious risk of bias	No serious inconsistency	No serious indirectness	No serious imprecision	None	321	311	–	SMD 0.79 lower (1.17 to 0.4 lower)	⊕⊕⊕⊕ HIGH	IMPORTANT
**ALT (better indicated by lower values)**												
9	Randomised trials	No serious risk of bias	No serious inconsistency	No serious indirectness	No serious imprecision	None	376	375	–	SMD 0.72 lower (1.01 to 0.44 lower)	⊕⊕⊕⊕ HIGH	IMPORTANT
**LDL-C (better indicated by lower values)**												
7	Randomised trials	No serious risk of bias	No serious inconsistency	No serious indirectness	No serious imprecision	none	267	263	–	SMD 0.53 lower (0.88 to 0.18 lower)	⊕⊕⊕⊕ HIGH	IMPORTANT
**HDL-C (better indicated by lower values)**												
4	Randomised trials	No serious risk of bias	No serious inconsistency	No serious indirectness	No serious imprecision	None	177	175	–	SMD 0.51 higher (0.12 lower to 1.15 higher)	⊕⊕⊕⊕ HIGH	IMPORTANT
**BMI (better indicated by lower values)**												
5	Randomised trials	No serious risk of bias	No serious inconsistency	No serious indirectness	No serious imprecision	none	215	205	–	SMD 0.58 lower (0.77 to 0.38 lower)	⊕⊕⊕⊕ HIGH	IMPORTANT
**TC (better indicated by lower values)**												
9	Randomised trials	No serious risk of bias	No serious inconsistency	No serious indirectness	No serious imprecision	none	367	357	–	SMD 0.74 lower (1 to 0.49 lower)	⊕⊕⊕⊕ HIGH	IMPORTANT
**HOMA-IR (better indicated by lower values)**												
5	Randomised trials	No serious risk of bias	No serious inconsistency	No serious indirectness	No serious imprecision	None^1^	241	231	–	SMD 1.56 lower (2.54 to 0.58 lower)	⊕⊕⊕⊕ HIGH	IMPORTANT

*CI* confidence interval, *SMD* Std. mean difference

### Strengths of this study

This meta-analysis pools data from several RCTs, resulting in a larger sample size. This larger pooled dataset enhances the statistical power of the analysis, allowing for a more precise estimation of the effects of berberine treatment on NAFLD. This is particularly advantageous for identifying small yet clinically significant differences that individual studies may be insufficiently powered to detect. By integrating findings from multiple studies, meta-analysis assists in addressing discrepancies and apparent contradictions observed in individual RCTs. This comprehensive approach helps elucidate whether observed variations are random, stem from methodological differences, or represent genuine heterogeneity in treatment effects. Additionally, through an in-depth observation of all included RCTs, four subgroup analyses were conducted to explore the sources of heterogeneity observed. Moreover, an analysis and summary of the safety of berberine were also performed.

Previous meta-analyses, such as the study by Ren [55], have focused on animal research. Basic research lays the groundwork for clinical studies, and when combining the findings from meta-analyses, a consistent pattern emerges in the effects of berberine on lipid profiles and other indicators in both animals and humans. However, built upon clinical trials, this study holds increased relevance for its potential clinical application. Importantly, this analysis underscores the multi-factorial benefits of berberine, not only in enhancing liver function but also in addressing metabolic dysfunctions associated with NAFLD. Our findings advocate for the inclusion of berberine in the therapeutic regimen for NAFLD, pending further research on its long-term benefits and safety profile.

### Limitations of this study

However, the potential of berberine as a treatment for NAFLD needs to be approached cautiously due to several notable limitations inherent in this meta-analysis. The study involved a relatively small cohort of 811 patients, mainly from trials conducted in China, hampering generalizability to other ethnic populations. Additionally, there was heterogeneity amongst the trials in optimal berberine dosage and duration of treatment. Several trials exhibited unclear or high risk of bias, undermining the reliability of the results. The brief treatment durations, ranging from 7 to 24 weeks, constrain the ability to draw conclusive insights regarding long-term efficacy and safety. Larger-scale RCTs, spanning more diverse populations and longer treatment periods, alongside metabolomic profiling, are essential for providing higher-quality evidence regarding the therapeutic value of berberine in NAFLD.

## Conclusions

This meta-analysis provides preliminary evidence that berberine may be an effective adjunct therapy for improving several metabolic parameters in patients with NAFLD. The mechanism behind the efficacy of berberine in treating NAFLD remains unclear. Yet, existing evidence indicates its potential as a therapeutic option for NAFLD.

### Supplementary Information

**Additional file 1: Table S1.** Literature search strategy.

**Additional file 2: Figure S1.** Subgroup analysis of ALT. **Figure S2.** Subgroup analysis of AST. **Figure S3.** Subgroup analysis of GGT. **Figure S4.** Subgroup analysis of TG. **Figure S5.** Subgroup analysis of TC. **Figure S6.** Subgroup analysis of LDL-C. **Figure S7.** Subgroup analysis of HDL-C. **Figure S8.** Subgroup analysis of HOMA-IR. **Figure S9.** Egger’s test and Begg’s test. **Figure S10.** Sensitivity analysis.

## Data Availability

The Supplementary Material includes the original contributions presented in the study; for further inquiries, please direct them to the corresponding author.

## Abbreviations

ALT: Alanine transaminase
AMPK: Adenosine monophosphate-activated protein kinase
AST: Aspartate transaminase
BMI: Body mass index
FXR: Farnesoid X receptor
GGT: Glutamyl transpeptidase
HDL-C: High-density lipoprotein cholesterol
HOMA-IR: Homeostasis model assessment for insulin resistance
InsR: Insulin receptor
IRS-1: Insulin receptor substrate 1
LDL-C: Low-density lipoprotein cholesterol
LSI: Lifestyle intervention;
NAFLD: Non-alcoholic fatty liver disease
Nrf2: Nuclear factor erythroid 2-related factor
PPAR: Peroxisome proliferation-activated receptor
RCT: Randomized controlled trial
ROS: Reactive oxygen species
ROS: Reactive oxygen species
SMD: Standardized mean difference
TC: Total cholesterol
TCA: Tricarboxylic acid
TG: Triglyceride
95% CI: 95% Confidence interval
