# Supplementary material for: The clinical efficacy and safety of berberine in the treatment of non-alcoholic fatty liver disease: a meta-analysis and systematic review
Source: J Transl Med. 2024 Mar 1;22:225. doi: 10.1186/s12967-024-05011-2 (PMC10908013; doi:10.1186/s12967-024-05011-2)
Supplement: Supplementary file 1 — Additional file 1: Table S1. Literature search strategy. [file 12967_2024_5011_MOESM1_ESM.docx]

# Table S1 Literature search strategy

**1.Pubmed**

| Search number | Query | Results |
| --- | --- | --- |
| #1 | **"****Berberine"[Mesh]** | 4273 |
| #2 | **"Non-alcoholic Fatty Liver Disease"[Mesh]** | 25378 |
| #3 | **((((((((nonalcoholic steatohepatitis[Title/Abstract]) OR (Non alcoholic Fatty Liver Disease[Title/Abstract])) OR (NAFLD[Title/Abstract])) OR (Nonalcoholic Fatty Liver Disease[Title/Abstract])) OR (Fatty Liver*, Nonalcoholic[Title/Abstract])) OR (Liver*, Nonalcoholic Fatty[Title/Abstract])) OR (Nonalcoholic Fatty Liver*[Title/Abstract])) OR (Nonalcoholic Steatohepatitis[Title/Abstract])) OR (Steatohepatitides, Nonalcoholic[Title/Abstract])** | 41547 |
| #4 | #2 or #3 | 43786 |
| #5 | #1 and #4 | 61 |

**2.Cochrane**

| Search number | Query | Results |
| --- | --- | --- |
| #1 | MeSH descriptor: [Non-alcoholic Fatty Liver Disease] explode all trees | 1835 |
| #2 | (nonalcoholic steatohepatitis):ti,ab,kw OR (Non alcoholic Fatty Liver Disease):ti,ab,kw OR (NAFLD):ti,ab,kw OR (Nonalcoholic Fatty Liver Disease):ti,ab,kw OR (Fatty Liver*, Nonalcoholic):ti,ab,kw | 4931 |
| #3 | (Liver*, Nonalcoholic Fatty):ti,ab,kw OR (Nonalcoholic Fatty Liver*):ti,ab,kw OR (Nonalcoholic Steatohepatitis):ti,ab,kw OR (Steatohepatitides, Nonalcoholic):ti,ab,kw | 4813 |
| #4 | #1 or #2 or #3 | 5054 |
| #5 | MeSH descriptor: [Berberine] explode all trees | 103 |
| #6 | #4 and #5 | 20 |

**3.Embase**

| Search number | Query | Results |
| --- | --- | --- |
| #1 | 'berberine'/exp | 10741 |
| #2 | 'nonalcoholic fatty liver'/exp OR 'nonalcoholic fatty liver' OR (nonalcoholic AND fatty AND ('liver'/exp OR liver)) OR 'nonalcoholic fatty liver disease':ab,ti OR 'nonalcoholic steatohepatitis':ab,ti OR 'non alcoholic fatty liver disease':ab,ti OR nafld:ab,ti OR 'fatty liver*, nonalcoholic':ab,ti OR 'liver, nonalcoholic fatty':ab,ti OR 'nonalcoholic fatty liver':ab,ti | 83336 |
| #3 | #1 and #2 | 175 |

**4.Web of science**

| Search number | Query | Results |
| --- | --- | --- |
| #1 | TS=(Berberine) | 17736 |
| #2 | (((((((((((TS=(nonalcoholic fatty liver disease))OR TS=(nonalcoholic fatty liver )) OR TS=(nonalcoholic fatty liver )) OR TS=(Non alcoholic Fatty Liver Disease)) OR TS=(NAFLD)) OR TS=(Nonalcoholic Fatty Liver Disease)) OR TS=(Fatty Liver*, Nonalcoholic)) OR TS=(Liver*, Nonalcoholic Fatty)) OR TS=(Nonalcoholic Fatty Liver*)) OR TS=(Nonalcoholic Steatohepatitis)) OR TS=(Steatohepatitides, Nonalcoholic)) OR TS=(Steatohepatitis, Nonalcoholic) | 28410 |
| #3 | #1 AND #2 | 130 |

**5.The Chinese National Knowledge Infrastructur**

| Search number | Query | Results |
| --- | --- | --- |
| #1 | 小檗碱 | 9370 |
| #2 | （主题：非酒精性脂肪肝）OR（主题：非酒精性脂肪性肝炎）OR（主题：非酒精性脂肪肝病）OR（主题：非酒精性脂肪性肝）OR（主题：非酒精性脂肪性肝病） | 27838 |
| #3 | #1 AND #2 | 85 |

**6.Wanfang Data**

| Search number | Query | Results |
| --- | --- | --- |
| #1 | 非酒精性脂肪肝 | 10105 |
| #2 | 小檗碱 | 8305 |
| #3 | #1 AND #2 | 34 |
