# Supplementary material for: The clinical efficacy and safety of berberine in the treatment of non-alcoholic fatty liver disease: a meta-analysis and systematic review
Source: J Transl Med. 2024 Mar 1;22:225. doi: 10.1186/s12967-024-05011-2 (PMC10908013; doi:10.1186/s12967-024-05011-2)

Figure S1 Subgroup analysis of ALT


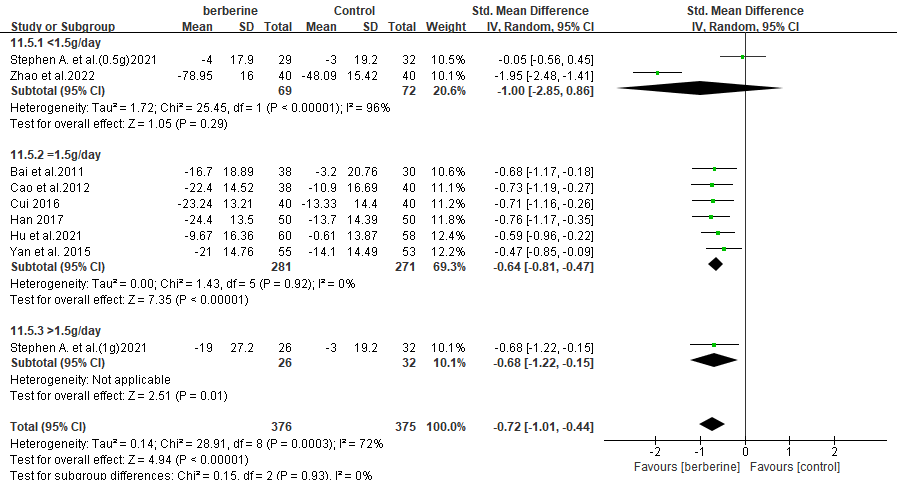

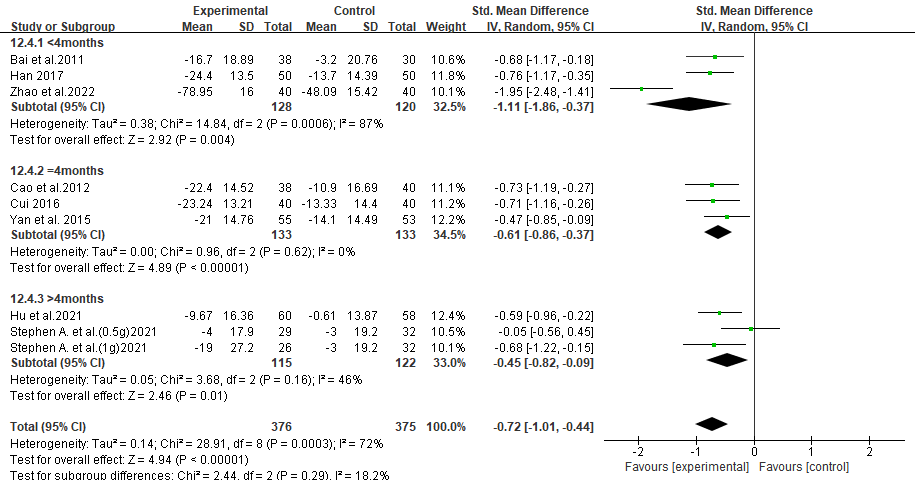

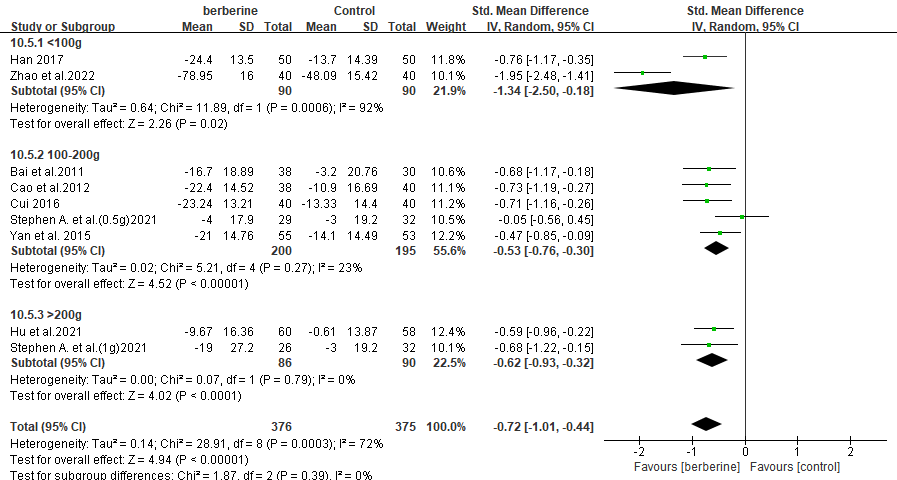

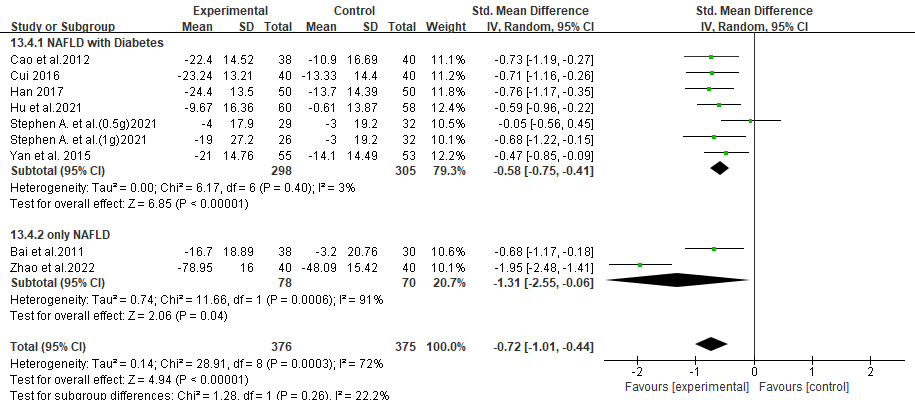


Figure S2 Subgroup analysis of AST


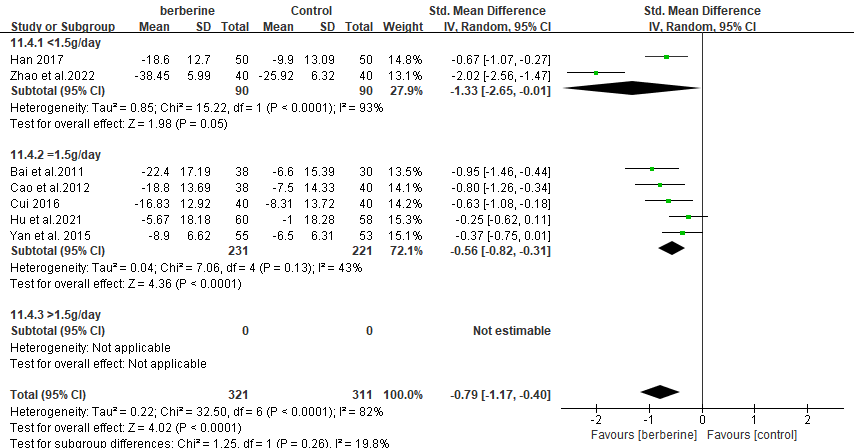

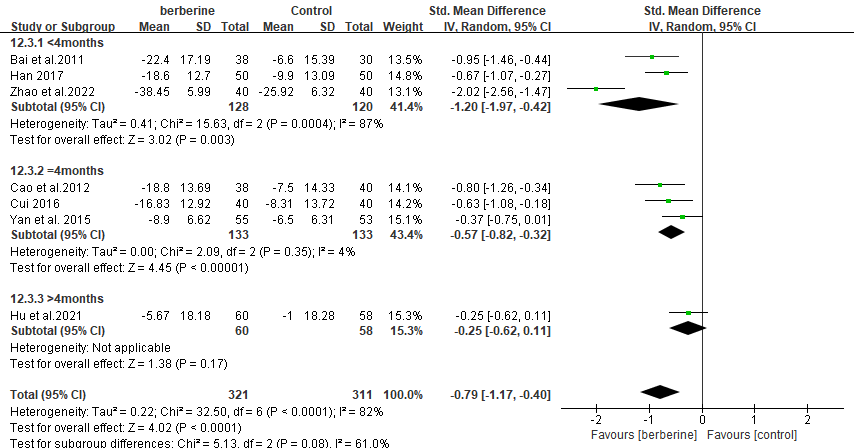

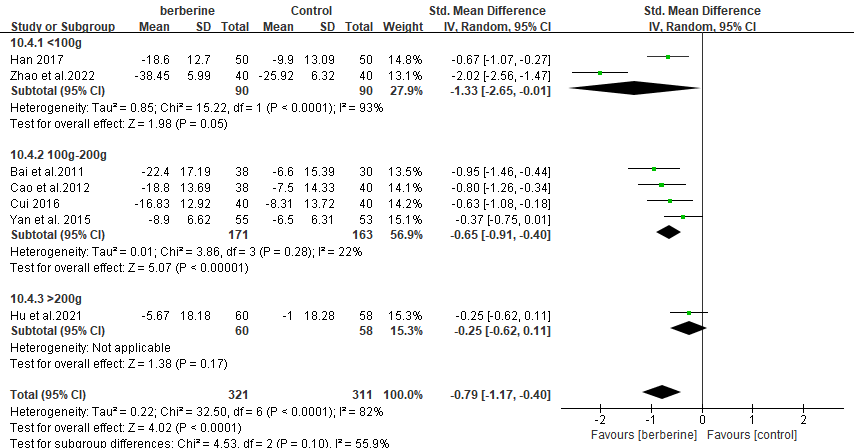

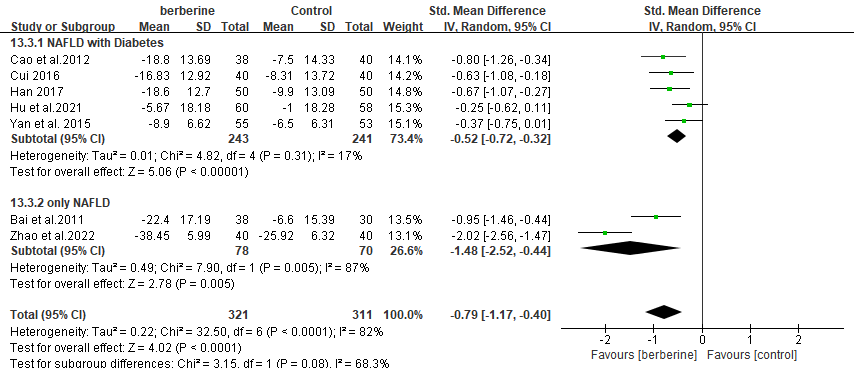


Figure S3 Subgroup analysis of GGT


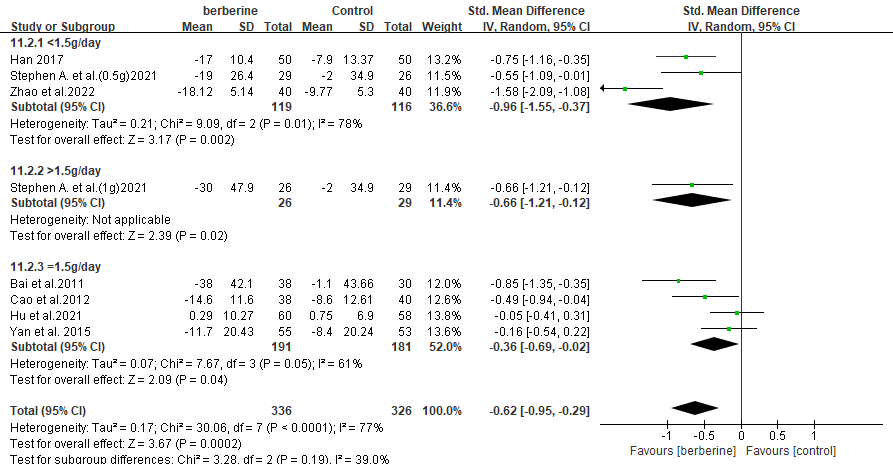

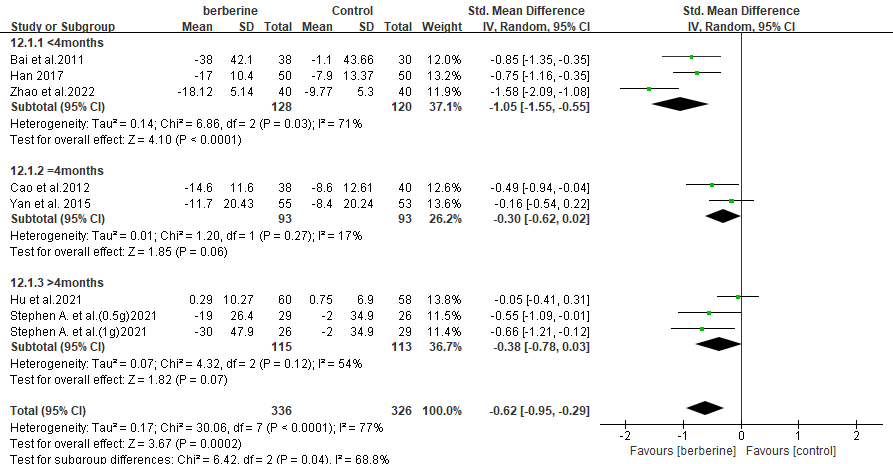

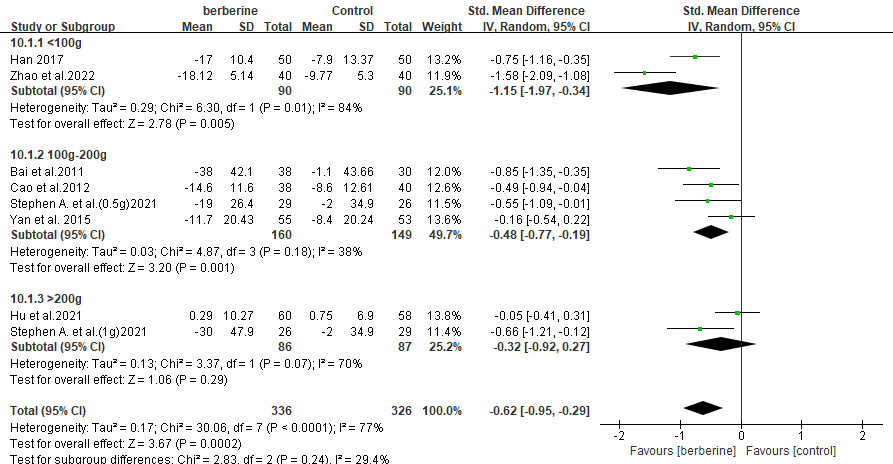

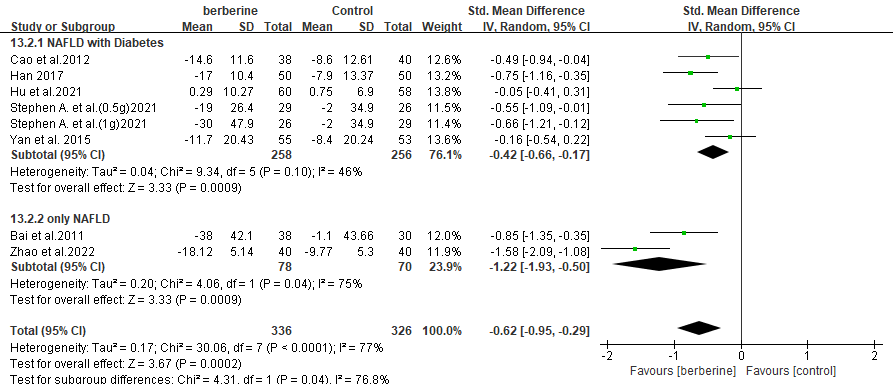


Figure S4 Subgroup analysis of TG


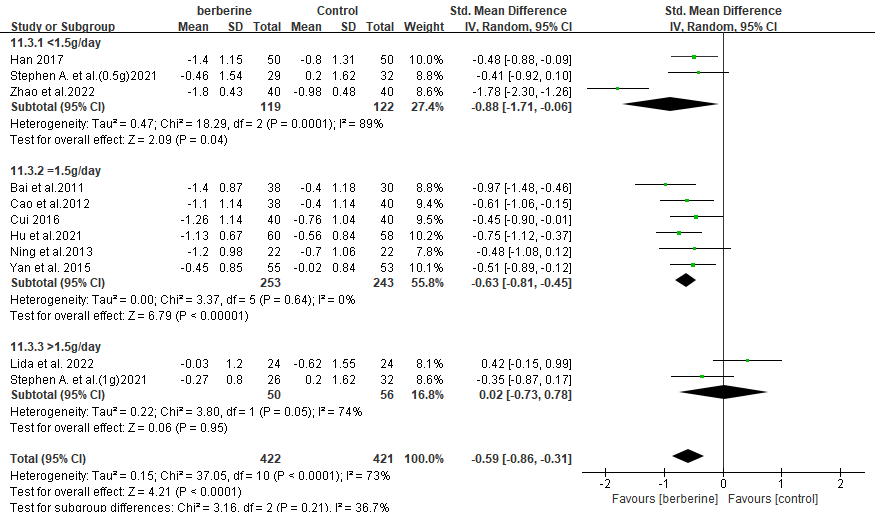

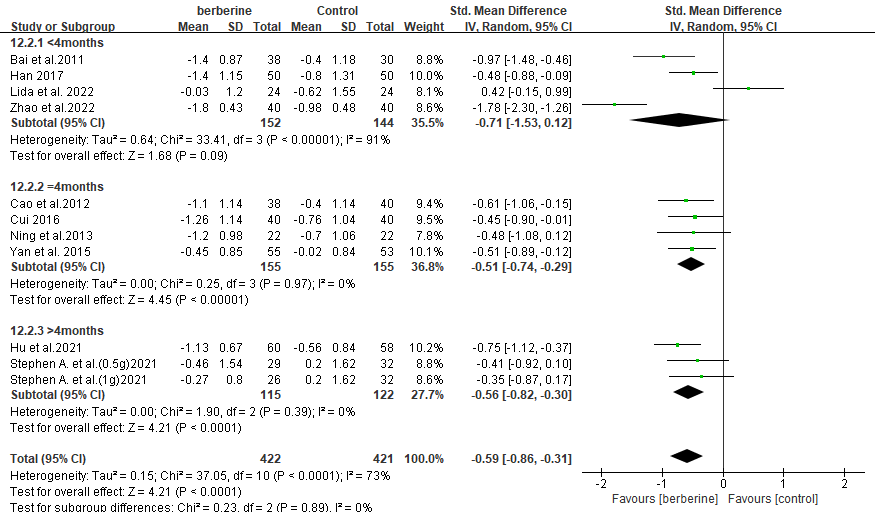

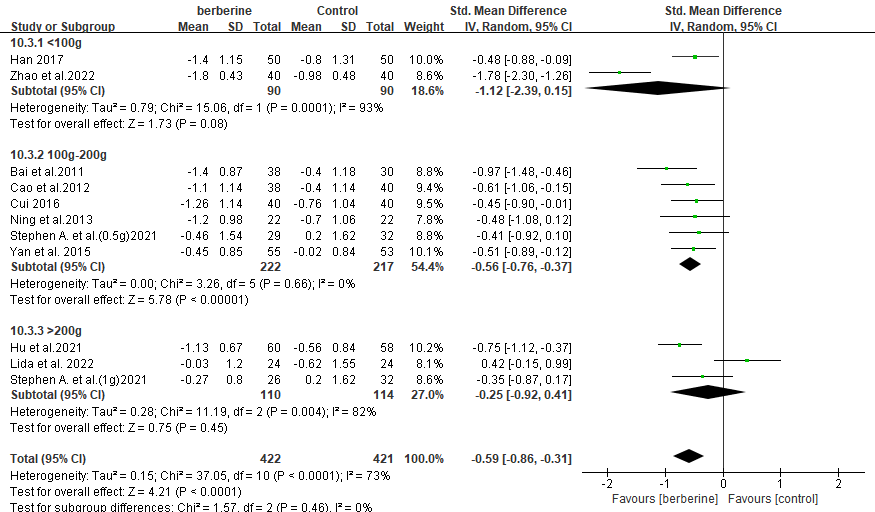

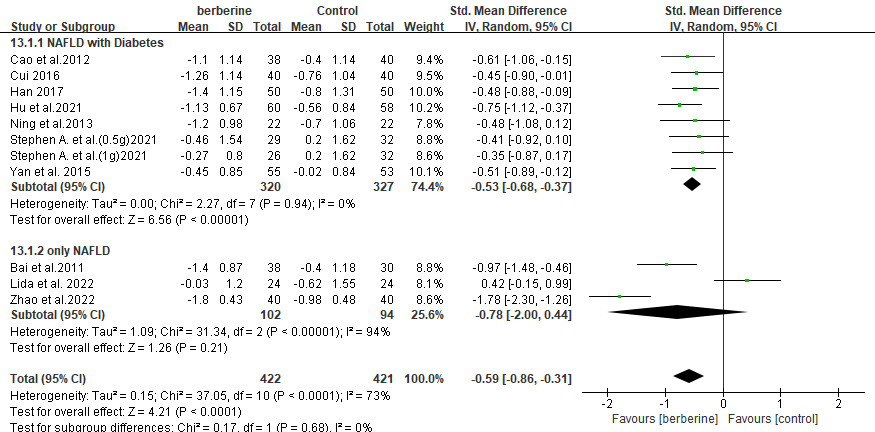


Figure S5 Subgroup analysis of TC


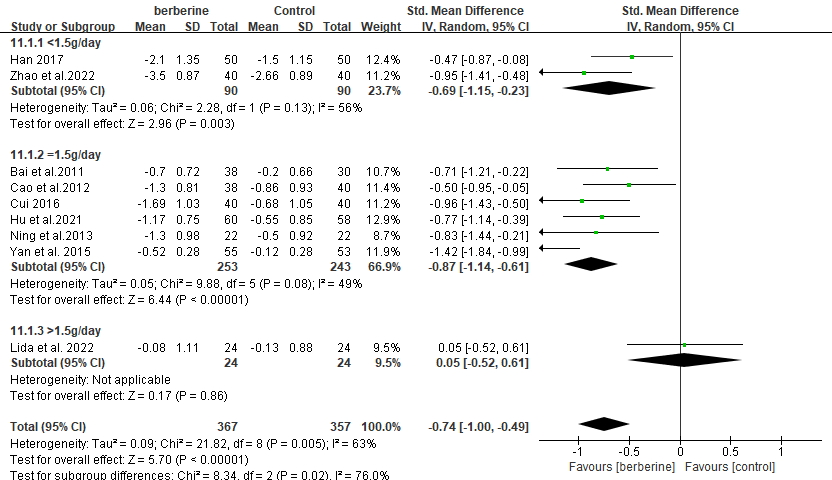

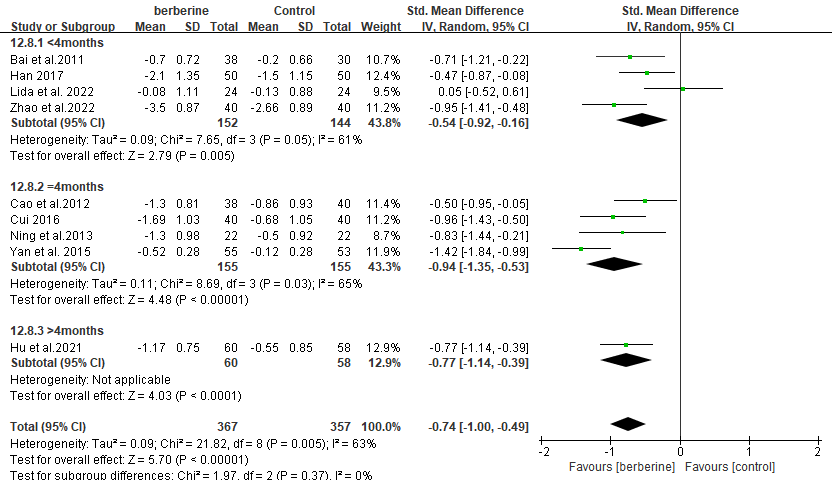

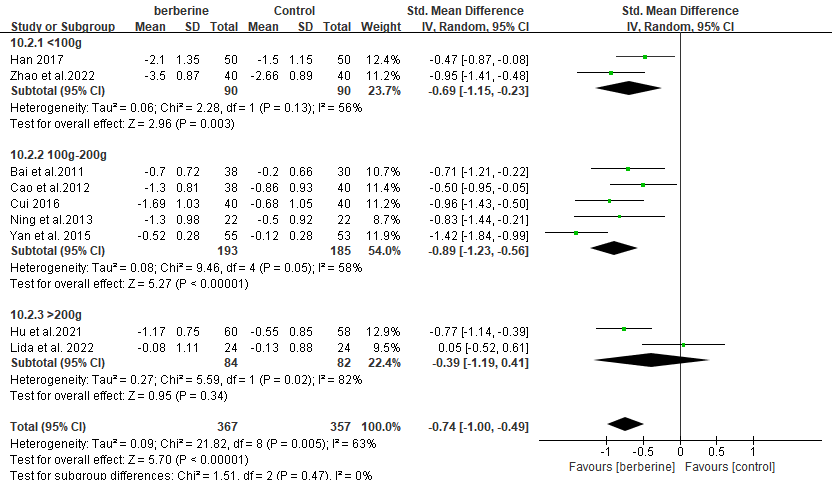

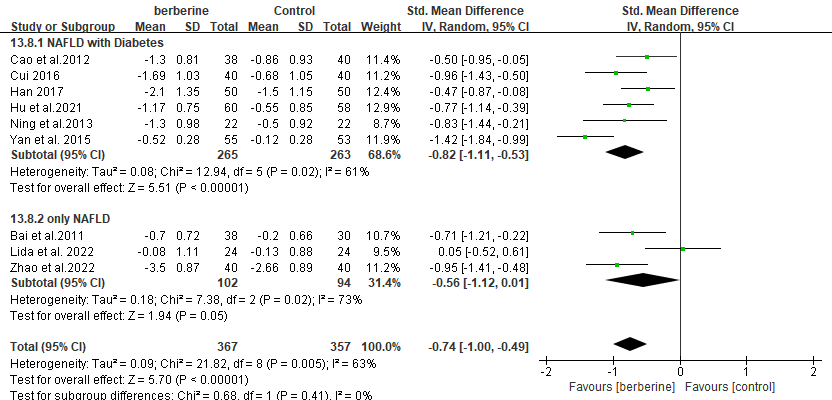


Figure S6 Subgroup analysis of LDL-C


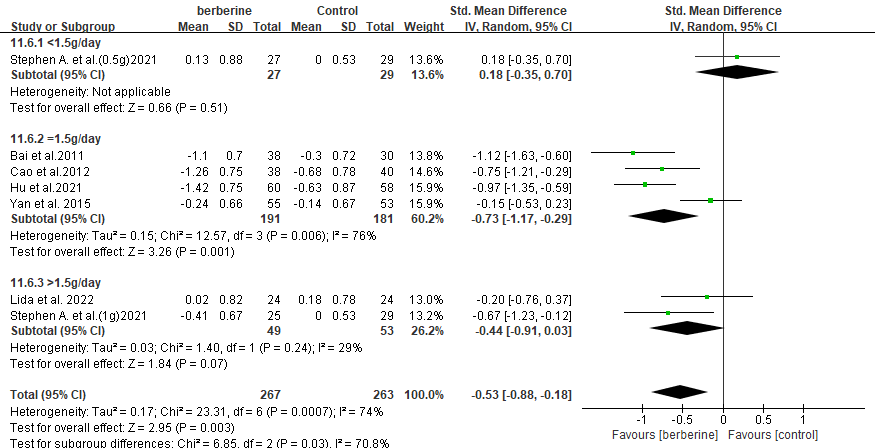

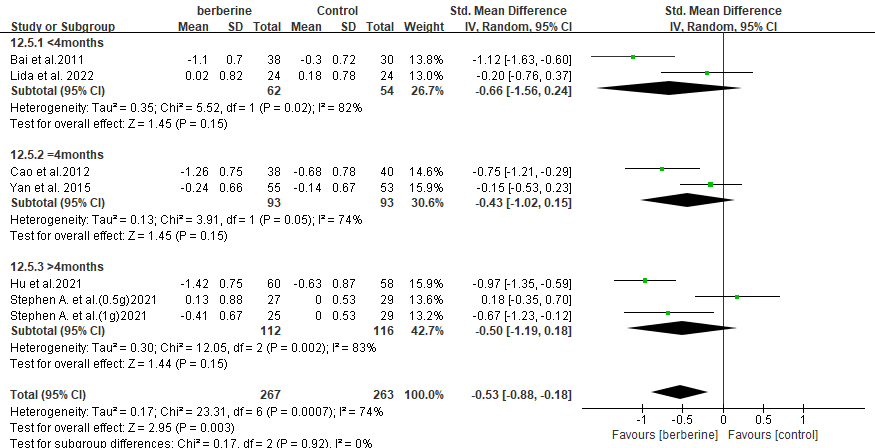

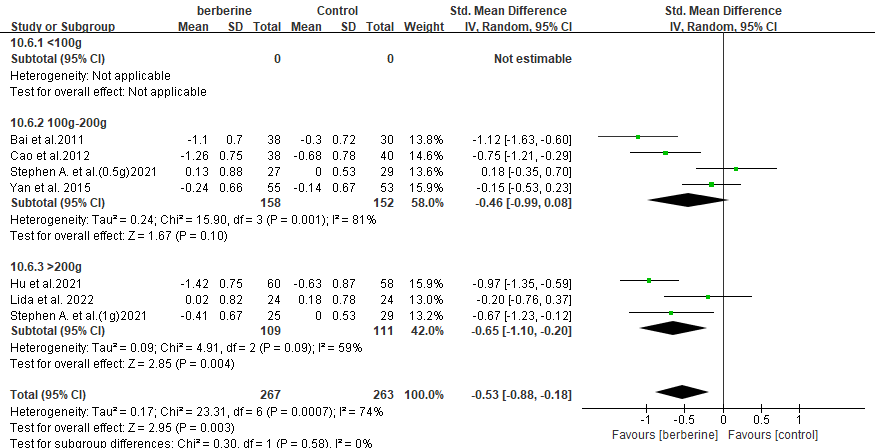




Figure S7 Subgroup analysis of HDL-C


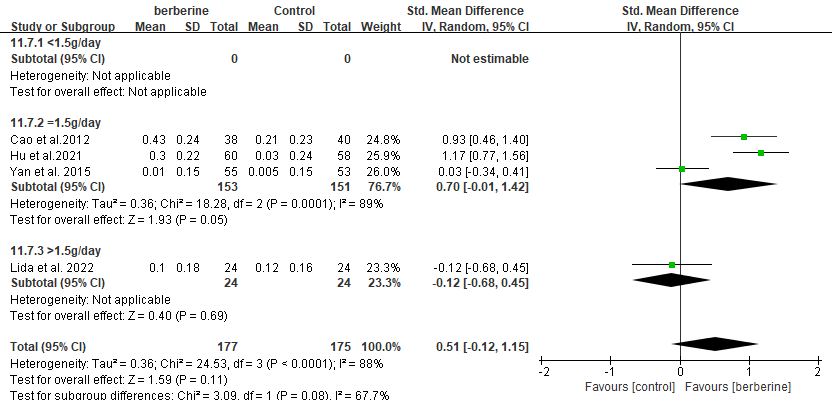

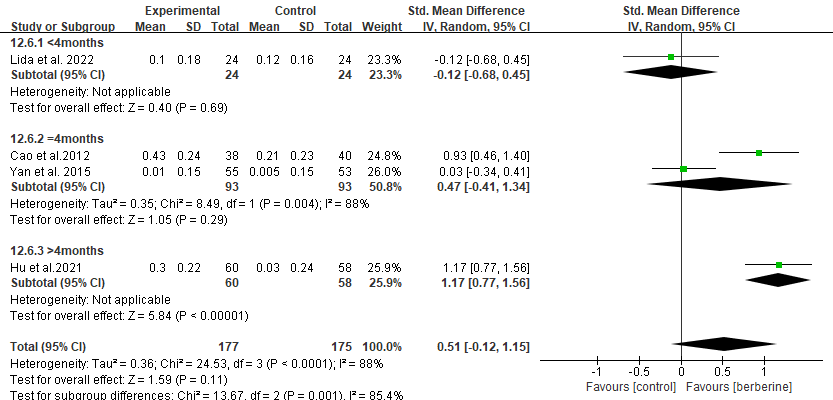

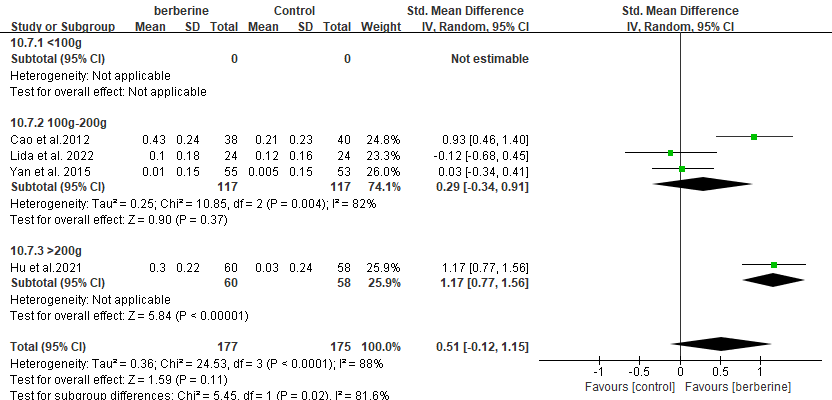

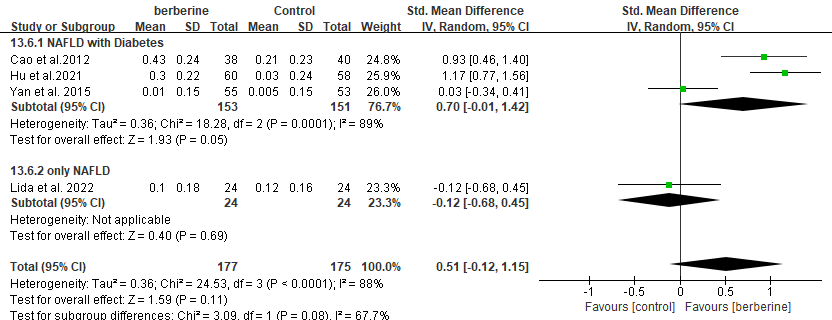


Figure S8 Subgroup analysis of HOMA-IR




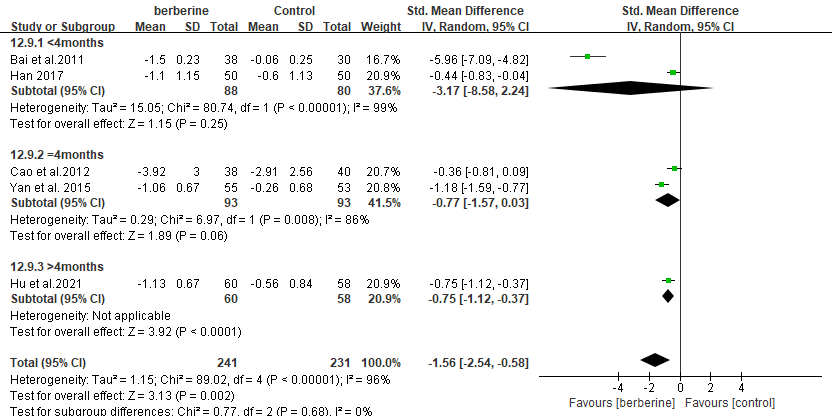

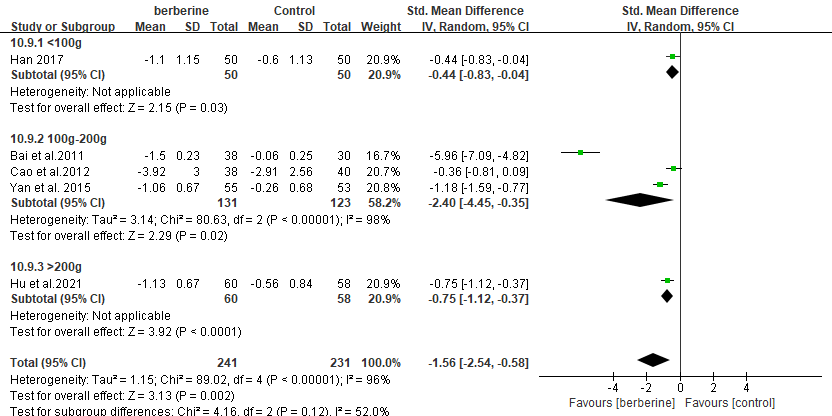




Figure S9 Egger's test and Begg's test


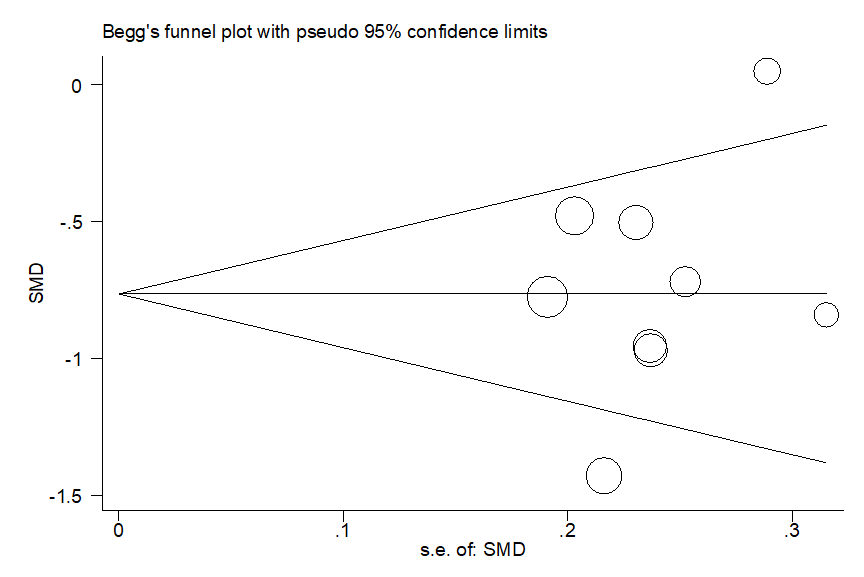

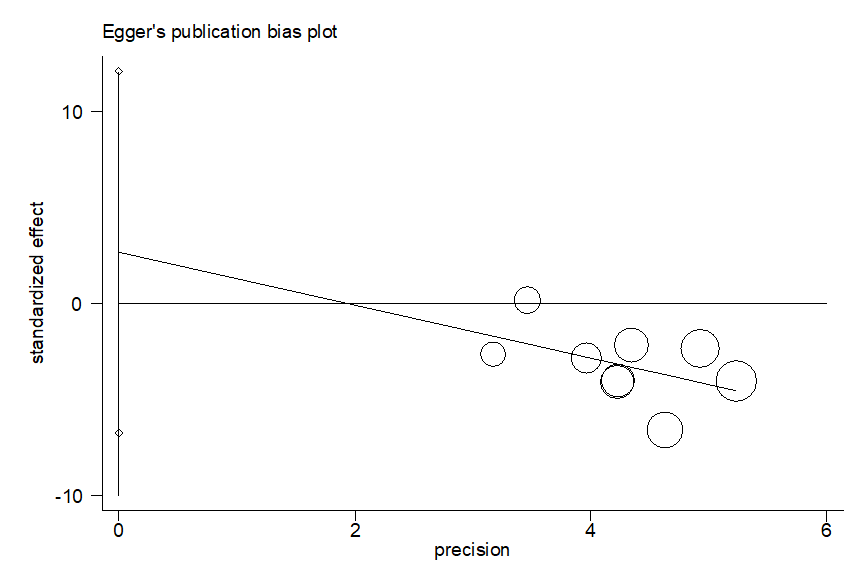


Figure S10 Sensitivity Analysis

Sensitivity Analysis of ALT


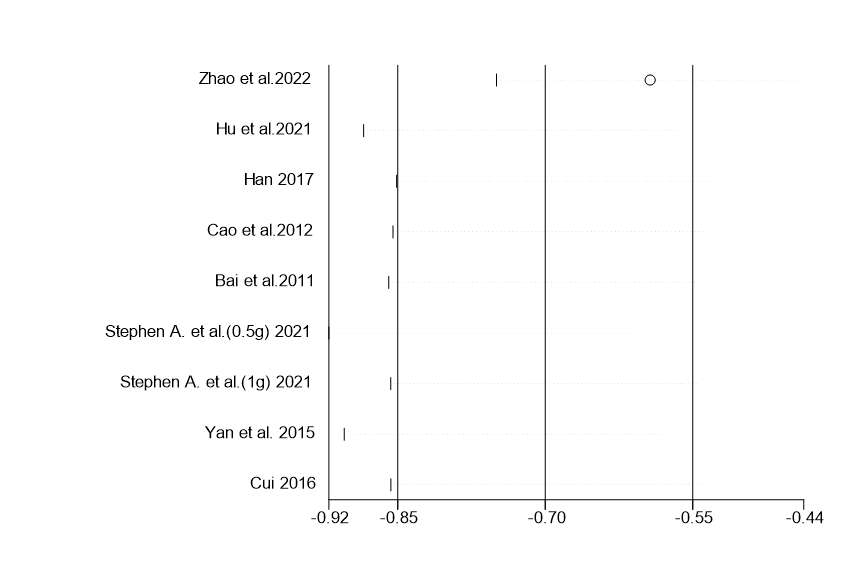


Sensitivity Analysis of AST


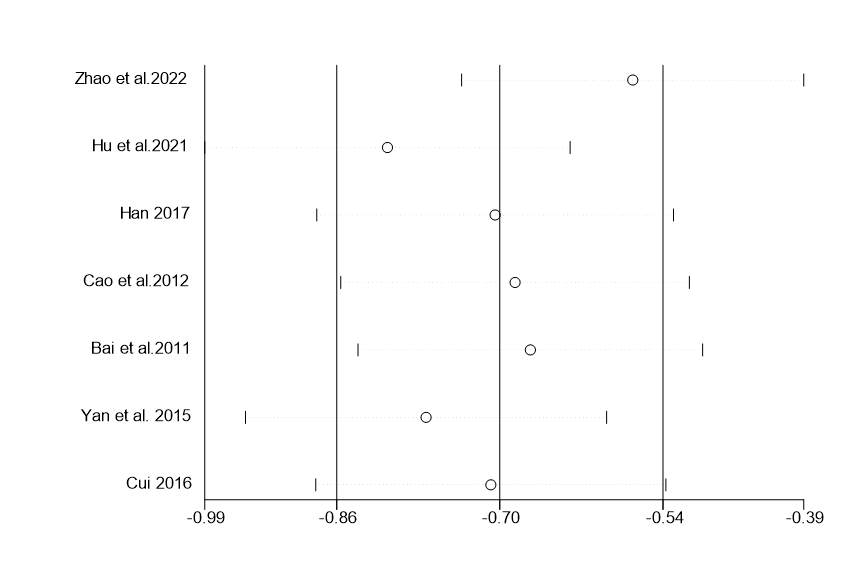


Sensitivity Analysis of BMI


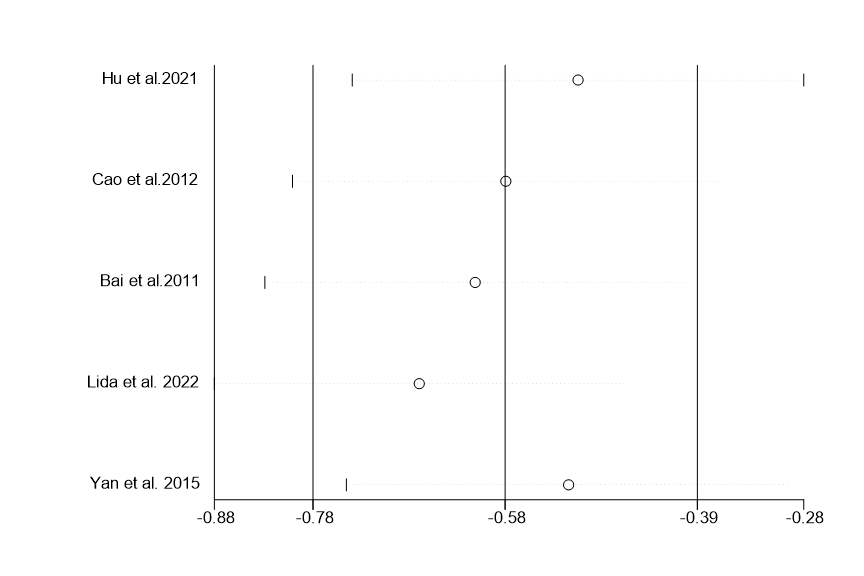


Sensitivity Analysis of GGT


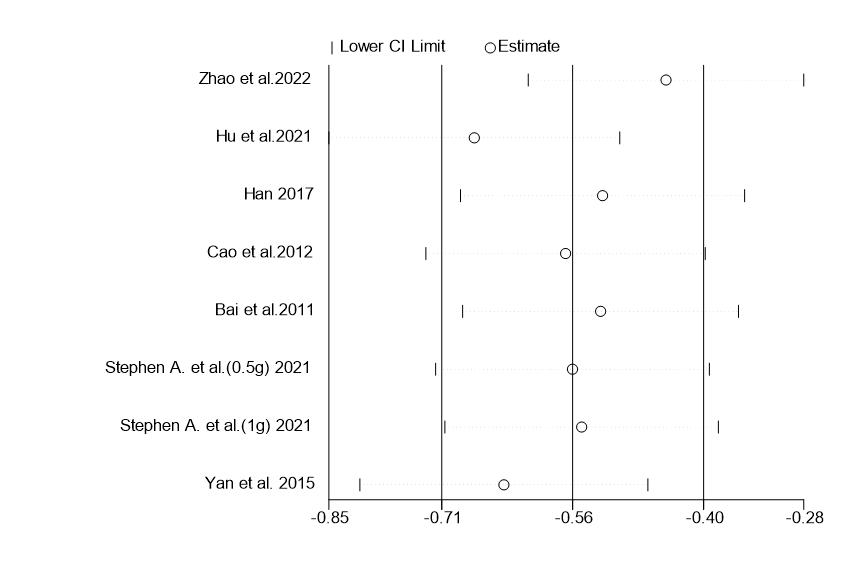


Sensitivity Analysis of HDL-C


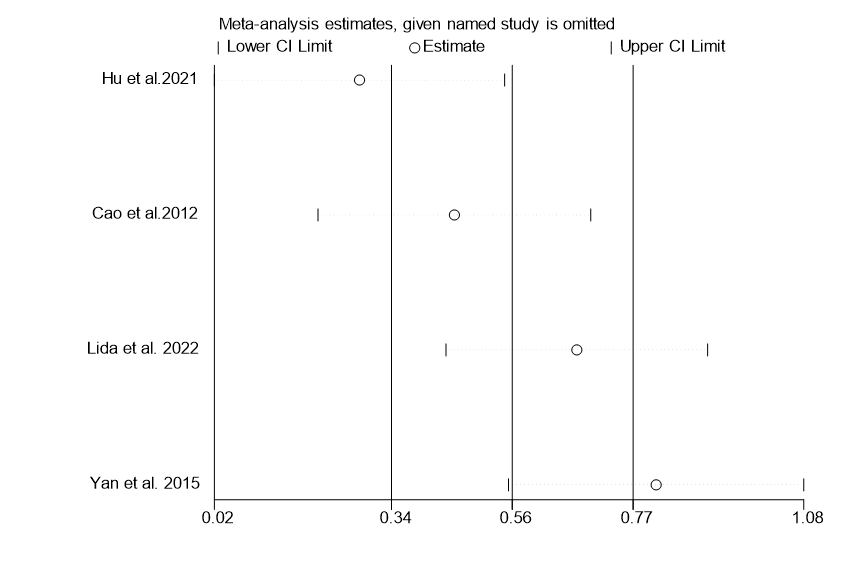


Sensitivity Analysis of HOMA-IR


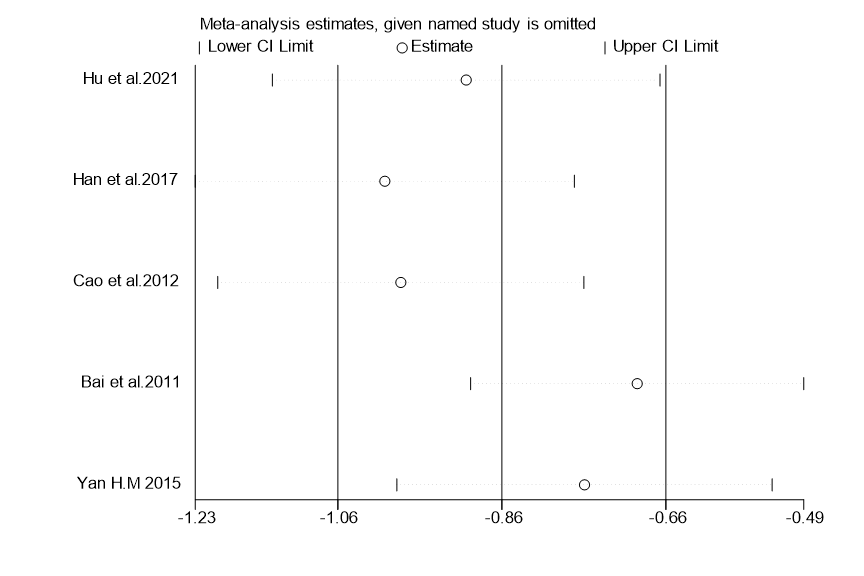


Sensitivity Analysis of LDL-C


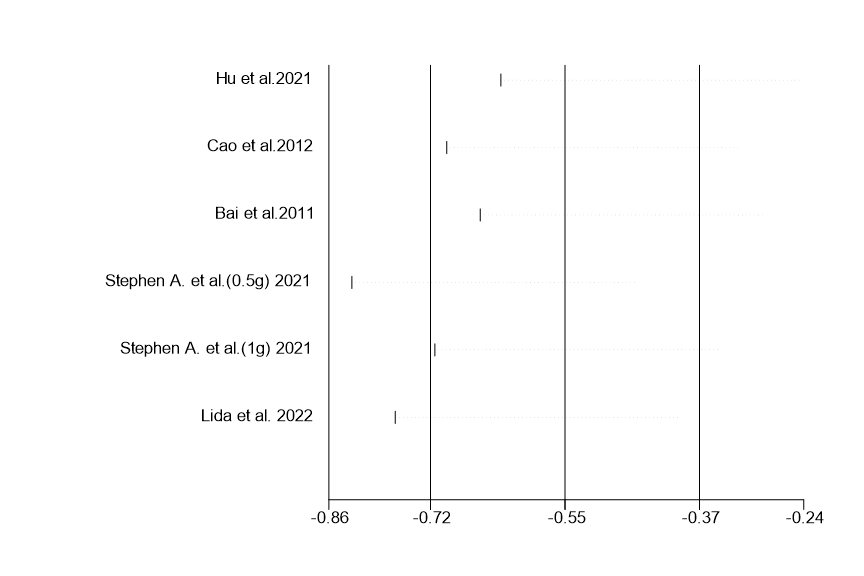


Sensitivity Analysis of TC


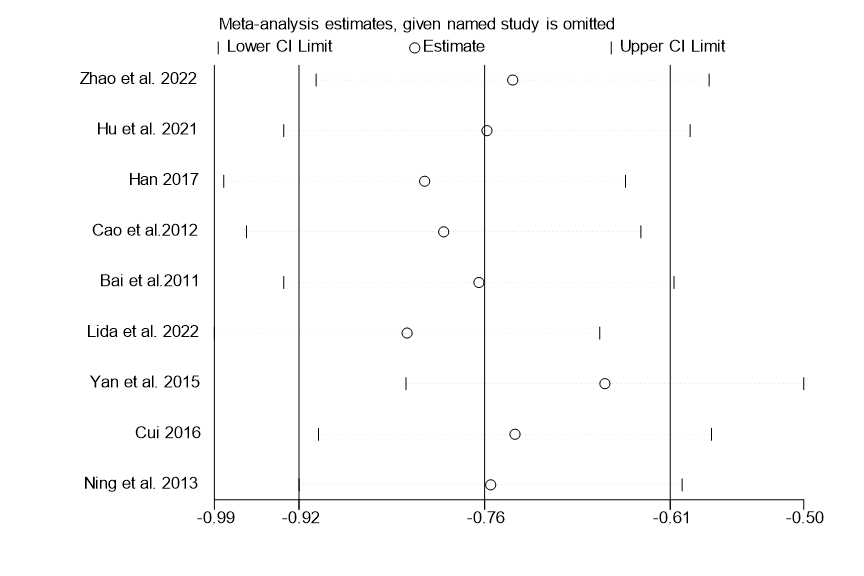


Sensitivity Analysis of TG


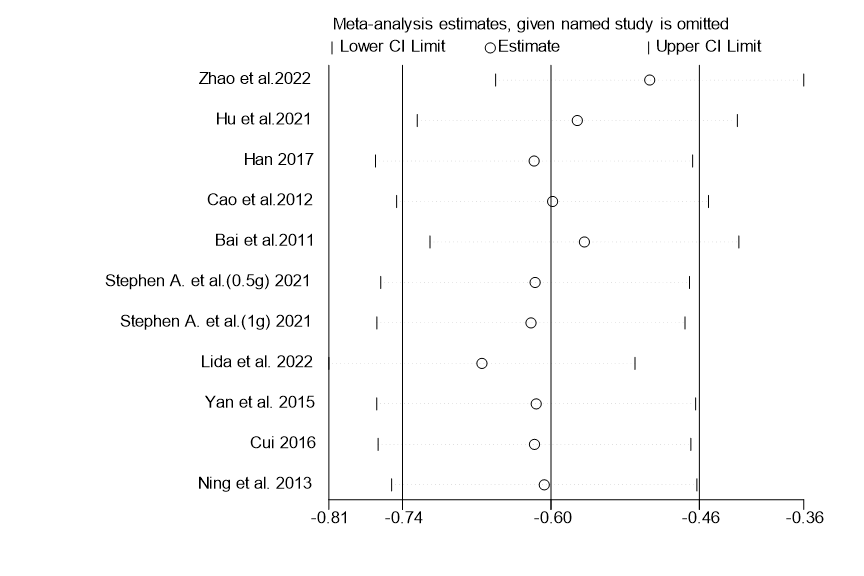

Supplement: Supplementary file 2 — Additional file 2: Figure S1. Subgroup analysis of ALT. Figure S2. Subgroup analysis of AST. Figure S3. Subgroup analysis of GGT. Figure S4. Subgroup analysis of TG. Figure S5. Subgroup analysis of TC. Figure S6. Subgroup analysis of LDL-C. Figure S7. Subgroup analysis of HDL-C. Figure S8. Subgroup analysis of HOMA-IR. Figure S9. Egger’s test and Begg’s test. Figure S10. Sensitivity analysis. [file 12967_2024_5011_MOESM2_ESM.docx]
